# GSTK1 suppresses HCC aggravation via L-carnitine metabolism by PGAM5/DRP1 complex-mediated mitochondrial quality control

**DOI:** 10.1186/s13046-025-03580-8

**Published:** 2025-11-24

**Authors:** Yuze Shi, Jinyao Zhang, Bojiao Song, Haitian Zhang, Jianbo He, Ke Ding, Fei Wang, Weiwei Yu, Guangyan Zhangyuan, Kangpeng Jin, Wenjie Zhang, Beicheng Sun

**Affiliations:** 1 Department of Hepatobiliary Surgery, The Affiliated Drum Tower Hospital of Nanjing University Medical School, Nanjing, Jiangsu Province 210008 China; 2 MOE Innovation Center for Basic Research in Tumor Immunotherapy, Hefei , Anhui Province 230022 China; 3 Anhui Province Key Laboratory of Tumor Immune Microenvironment and Immunotherapy, Hefei, Anhui Province 230022 China; 4 Department of Hepatobiliary Surgery, The First Affiliated Hospital of Anhui Medical University, Hefei, Anhui Province 230022 China; 5 Department of Thoracic and Cardiovascular Surgery, The Affiliated Drum Tower Hospital of Nanjing University Medical School, Nanjing, Jiangsu Province 210008 China; 6 Department of General Surgery, Ruijin Hospital, Shanghai Jiao Tong University School of Medicine, Shanghai, 200025 China; 7 Department of General Surgery, Colorectal Institute of Nanjing Medical University, The First Affiliated Hospital of Nanjing Medical University, Nanjing, Jiangsu Province 210029 China

**Keywords:** Glutathione s-transferase kappa 1, Hepatocellular carcinoma, Mitochondrial quality control, L-carnitine, Phosphoglycerate mutase 5

## Abstract

**Background:**

Hepatocellular carcinoma (HCC) is among the leading causes of cancer-related mortality worldwide. The high recurrence rate and resistance to chemotherapy of HCC contribute to poor clinical outcomes, necessitating the development of novel therapeutic strategies. Glutathione S-transferase kappa 1 (GSTK1) is specifically localized to mitochondria and peroxisomes, participates in adiponectin secretion and insulin resistance, and inhibits the progression of non-alcoholic fatty liver disease. However, the role of GSTK1 in HCC is unknown. We aimed to determine the role of GSTK1 in HCC progression.

**Methods:**

N-nitrosodiethylamine (DEN)/ carbon tetrachloride and DEN/high-fat, high-fructose, high-cholesterol diet models were used in hepatocyte-specific Gstk1 knockout and control mice to establish a murine HCC model. Human HCC cell lines with GSTK1 overexpression or knockdown were used to determine GSTK1 function in tumor growth and migration in vitro. Non-target metabolomics analysis, RNA-sequence, transmission electron microscope (TEM), immunoprecipitation (IP), liquid chromatography, and high-throughput mass spectrometry (LC-MS/MS) were used to determine the mechanism by which GSTK1 participates in HCC.

**Results:**

GSTK1 was shown to suppress HCC in vivo and in vitro. Non-target metabolomics analysis indicated that GSTK1 participates in L-carnitine metabolism. L-carnitine supplementation inhibited proliferation and promoted apoptosis of HCC cells in vivo and in vitro. This effect was enhanced by GSTK1 overexpression. Mechanically, TEM and western blot showed that GSTK1 influences mitochondrial quality control (MQC) by promoting mitochondrial biosynthesis and mitochondrial fusion. GSTK1 was shown to inhibit mitochondrial fission and mitophagy, which was consistent with the immunofluorescence results. IP and LC-MS/LMS indicated that GSTK1 combines with PGAM5 and competes with DRP1. Additionally, GSTK1 was shown to be regulated by transcription factors (PPARα/RXRα) and the RXRα agonist, bexarotene, inhibited HCC cell proliferation.

**Conclusions:**

GSTK1 was shown to be a tumor suppressor via its role in MQC and L-carnitine metabolism. Bexarotene and L-carnitine supplementation may serve as potential therapeutic strategies for HCC treatment.

**Graphical abstract:**

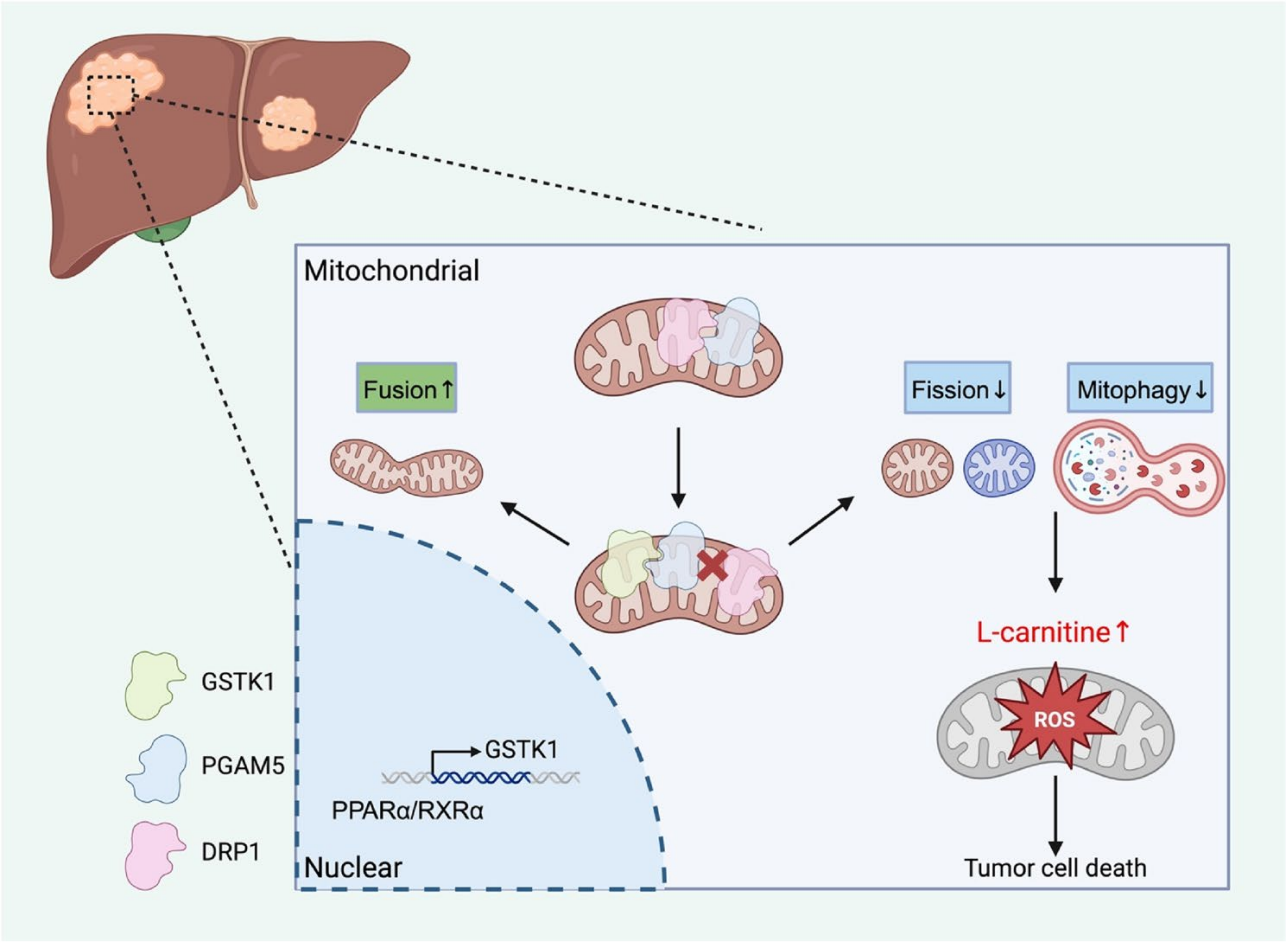

**Supplementary Information:**

The online version contains supplementary material available at 10.1186/s13046-025-03580-8.

## Introduction

Hepatocellular carcinoma (HCC) ranks as the third leading cause of cancer-related mortality worldwide and is primarily driven by a combination of genetic, epigenetic, and environmental factors [1]. HCC is mainly caused by exposure to carcinogens, hepatitis virus infection (hepatitis B virus [HBV] or hepatitis C virus [HCV]), and metabolic liver diseases [2]. Notably, non-alcoholic steatohepatitis (NASH), recently redefined as metabolic dysfunction-associated steatohepatitis (MASH), represents a major risk factor for both cirrhosis and HCC, with its incidence rising steadily in recent years [3].

Over the same period, cancer has been increasingly recognized as a metabolic disorder with key metabolic pathways, such as carbohydrate, amino acid, nucleotide, fatty acid, and lipid metabolism, being remodeled [4]. HCC cells undergo significant metabolic reprogramming to support rapid growth and survival [5]. However, progress in targeting cancer metabolism therapeutically in the past decade has been limited. Only a few metabolism-based drugs for cancer have been successfully developed, some of which are currently undergoing or awaiting clinical trials. Recent findings have suggested that the carnitine system (CS) could be considered as a gridlock to finely trigger the metabolic flexibility of cancer cells [6]. Carnitine metabolism is achieved through the carnitine palmitoyl transferase system (CPTS), which mainly consists of carnitine palmitoyl-transferase (CPT)1, CPT2, and carnitine/acyl-carnitine translocase (CACT). Metabolic enzymes regulate metabolic plasticity through the CS, which suggests use of metabolic enzymes for developing new therapeutic strategies. For example, CPT1A is an appealing druggable target for cancer therapies because CPT1A is essential for the survival, proliferation, and drug resistance of cancer cells [7]. Moreover, CPT1A lowers the risk of cancer recurrence and metastasis, reduces mortality, and offers prospective therapy options for clinical treatment if the effects of CPT1A on the lipid metabolism of cancer cells are inhibited [8].

Carnitine metabolism mainly occurs in the mitochondria, which are considered the bioenergetic, biosynthetic, and signaling center of the cell [9]. To safeguard the mitochondria against damage and prevent the accumulation of defective mitochondria, the body achieves a multifaceted group of processes (mitochondrial quality control [MQC]). Such processes mainly involve three distinct mechanisms (mitochondrial biogenesis, mitochondrial dynamics, and mitophagy). Peroxisome proliferator-activated receptor gamma coactivator (PGC)−1α, a master regulator of mitochondrial biogenesis, is involved in the metabolic reprogramming process of cancer progression. PGC-1α inhibits the proliferation and survival of cancer cells, and low PGC-1α expression may be associated with the malignant progression and poor prognosis of HCC [10]. Mitochondrial dynamics mainly refers to the dynamic interplay between fission and fusion, which determine the morphology, quality, quantity, and distribution of mitochondria within cells, as well as mitochondrial function [11]. Cancer cells often exhibit fragmented mitochondria. High expression or enhanced activation of DRP1 and/or downregulation of MFN2 mediate this phenotype in colorectal, lung, and pancreatic cancer. Enhanced fission or reduced fusion is related to poor prognosis of HCC [12]. Mitophagy involves the process of selectively targeting mitochondria by autophagosomes for mitochondrial degradation. Notably, mitophagy and mitochondrial biogenesis are two opposing physiologic processes. Given the complex and heterogeneous nature of cancer, coupled with the multifaceted effects of mitophagy on tumor biology, the specific role of mitophagy in different cancers and cancer stages, remains largely unknown. Inhibition and induction of mitophagy are strategies worth considering in cancer treatment [13].

Glutathione S-transferase kappa 1 (GSTK1), also known as disulfide-bond A oxidoreductase-like protein (DsbA-L), is located in mitochondria and peroxisomes [14]. Previous studies demonstrated that DsbA-L promotes the multimerization of adiponectin and prevents oxidative stress damage, obesity-induced inflammation, and insulin resistance [15, 16]. However, it is unknown whether *GSTK*1 regulates HCC progression. Herein hepatocyte *Gstk1* was shown to suppress tumorigenesis by regulating L-carnitine metabolism, which functions against HCC. Mechanistically, GSTK1 could prevent combining PGAM5 with DRP1, which inhibits mitochondrial fission and mitophagy.

## Results

### Hepatocyte *Gstk1* loss promotes HCC progression

**Fig. 1:**
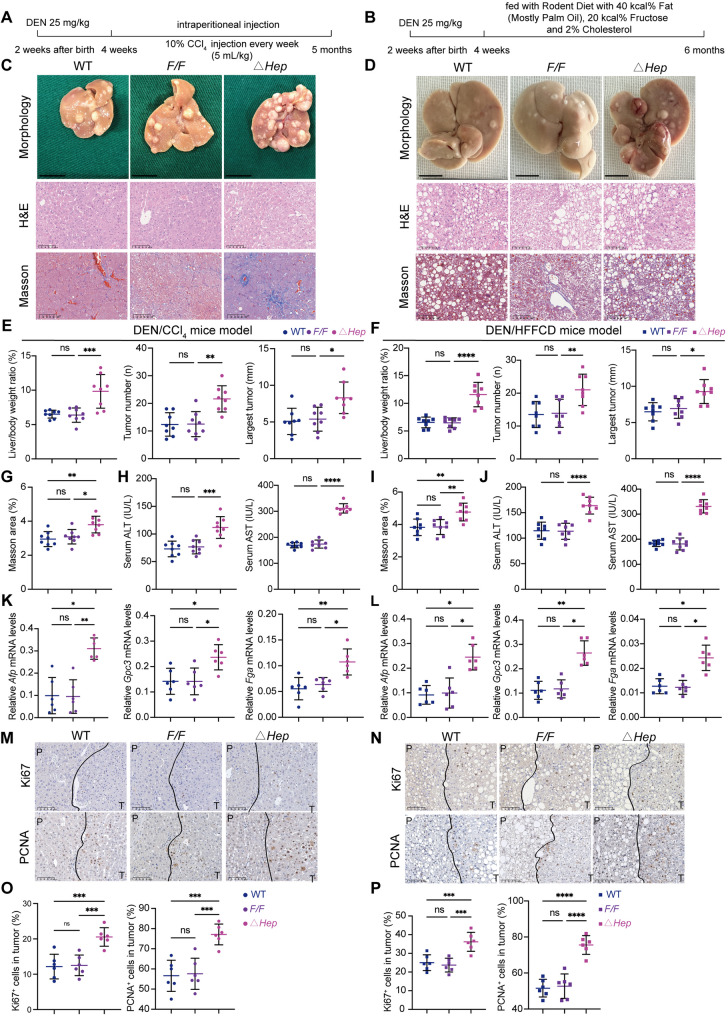
Hepatocyte *GSTK1* loss promotes DEN/CCl_4_- or DEN/HFFCD-induced murine HCC model. (**A**-**B**) Schematic diagram of DEN/CCl_4_- or DEN/HFFCD-induced HCC model. (**C**-**D**) Representative images of liver morphology, and H&E and Masson staining in liver sections from DEN/CCl_4_- or DEN/HFFCD-treated mice (bar = 100 μm). (**E**-**F**) Statistical analysis of liver weight: body weight ratio, tumor number, and largest tumor (*n* = 8). (**G** and **I**) Statistical analysis of Masson staining of liver sections from **C**, **D** (*n* = 8). (**H** and **J**) Serological analysis of AST and ALT of DEN/CCl_4_- or DEN/HFFCD-treated mice (*n* = 8). (**K**-**L**) RT-qPCR analysis of HCC markers in DEN/CCl_4_- or DEN/HFFCD-treated mice (*n* = 6). (**M**-**P**) Representative images and statistical analysis of Ki67 and PCNA staining of liver sections from DEN/CCl_4_- or DEN/HFFCD-treated mice (*n* = 6; bar = 100 μm). Data are presented as the mean ± SD. n.s, no significance, * *P* < 0.05, ** *P* < 0.01, and *** *P* < 0.001, and **** *P* < 0.0001

Hepatocyte-specific *Gstk1* deletion mice (*Gstk1*^*△Hep*^) were generated and the deletion of Gstk1 in hepatocytes was confirmed by polymerase chain reaction (PCR) and immunoblotting analysis (Figure S1A-C). Then, DEN/CCl_4_ and DEN/HFFCD murine models were employed to determine the role of hepatocyte *Gstk1* in HCC (Fig. 1A, B). All mice in the DEN/HFFCD murine model developed HCC with typical MAFLD characteristics, as analyzed by the entire liver morphology, hematoxylin and eosin staining, and Masson trichrome staining (Fig. 1C-D, G, I). Notably, the liver weight-to-body weight ratio, maximum tumor diameter, and tumor load were more severe in *Gstk1*^*△Hep*^ mice when compared to *Gstk1*^*Flox/Flox*^ *(Gstk1*^*F/F*^*)* mice or wild-type (*WT)* littermates (Fig. 1E, F). Similar results were obtained when the HCC markers (*Afp*, *Gpc3*, and *Fga*) were examined by RT-qPCR (Fig. 1K, L). Serum ALT and AST were measured in all mice; *Gstk1*^*△Hep*^ mice had the highest ALT and AST levels in both models, suggesting more severe liver dysfunction (Fig. 1H-J). Additionally, immunohistochemical analysis revealed increased labeling of proliferating cell nuclear antigen (PCNA) and Ki67 in *Gstk1*^*△Hep*^ group (Fig. 1M-P).

These findings demonstrate that hepatocyte-specific loss of Gstk1 increases susceptibility to chemically induced or MASH-driven HCC and promotes tumor proliferation.

### GSTK1 inhibits the proliferation and migration of HCC cell lines

**Fig. 2:**
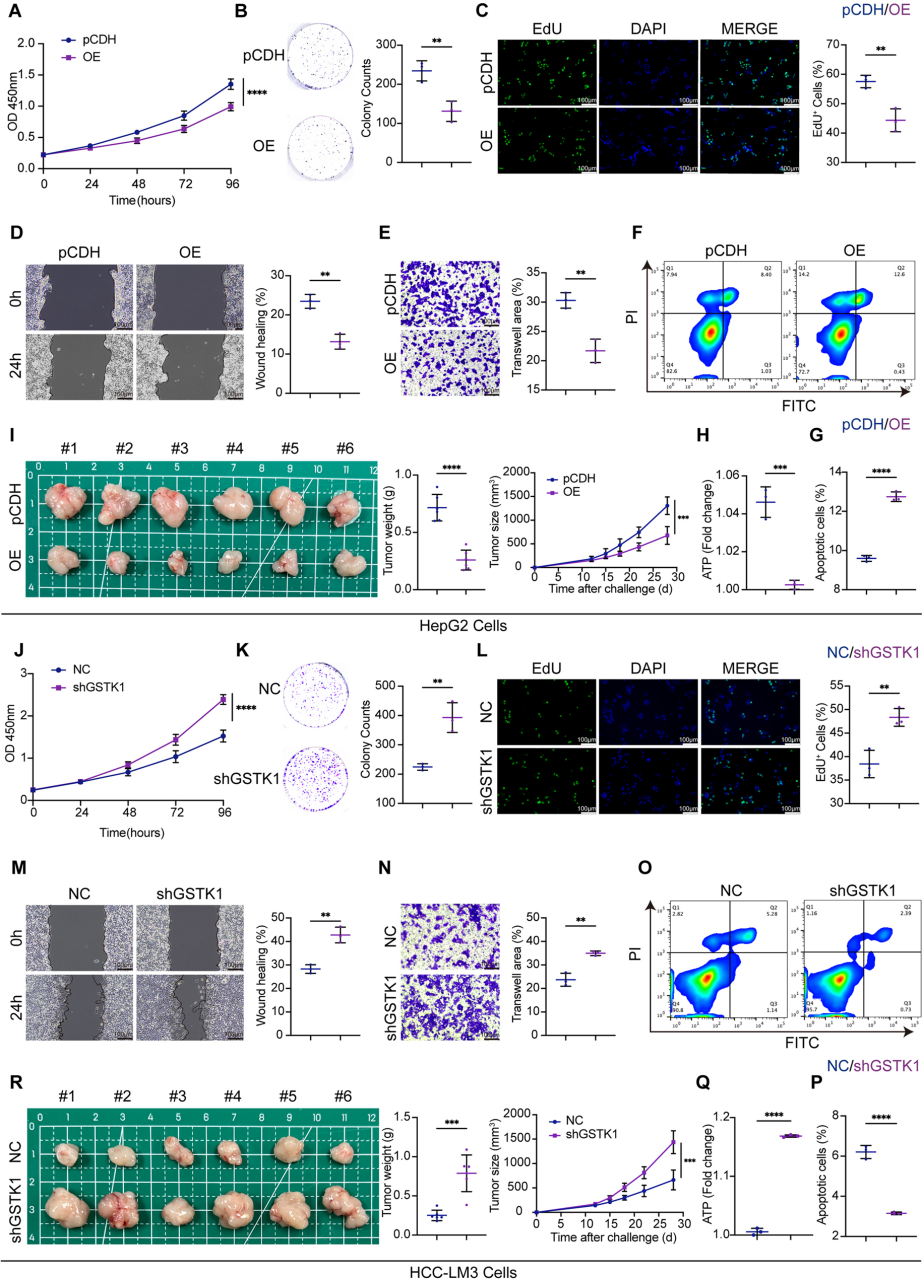
GSTK1 inhibited proliferation and migration in HCC cell lines. (**A-C**) Cell proliferation ability of HepG2 cells after GSTK1 overexpression. CCK8 (**A**), colony formation (**B**), EdU (**C**) bar = 100 μm. (**D-E**) Cell migration ability of HepG2 cells after GSTK1 overexpression. Wound healing (**D**), Transwell (**E**) bar = 100 μm. (**F** and **G**) Apoptotic ratio of HepG2 cells after GSTK1 overexpression detected by flow cytometry using the Annexin V-FITC/PI staining kit (**F**), statistical analysis (**G**). (**H**) ATP measurement of HepG2 cells after GSTK1 overexpression. (**I**) HepG2 cells with GSTK1 overexpression were transplanted on BALB/c-Nude mice; tumor volumes and tumor weights were recorded (*n* = 6). (**J-L**) Cell proliferation ability of HCC-LM3 cells after GSTK1 knockdown. CCK8 (**J**), colony formation (**K**), EdU (**L**) bar = 100 μm. (**M-N**) Cell migration ability of HCC-LM3 cells after GSTK1 knockdown. Wound healing (**M**), Transwell (**N**) bar = 100 μm. (**O** and **P**) Apoptotic ratio of HCC-LM3 cells after GSTK1 knockdown detected by flow cytometry using the Annexin V-FITC/PI staining kit (**O**), statistical analysis (**P**). (**Q**) ATP measurement of HCC-LM3 cells after GSTK1 knockdown. (**R**) HCC-LM3 cells with GSTK1 knockdown were transplanted on BALB/c-Nude mice. Tumor volumes and tumor weights were recorded (*n* = 6). Data are presented as the mean ± SD. n.s, no significance, * *P* < 0.05, ** *P* < 0.01, *** *P* < 0.001, and **** *P* < 0.0001

GSTK1 overexpression was used in HepG2 and Hep3B cells, and GSTK1 knockdown was used in HCC-LM3 and Huh7 cells to determine the functional role of GSTK1 in HCC in vitro (Figure S1D, E). In vitro assays, including colony formation, 5-Ethynyl-2’-deoxyuridine (EdU), and CCK-8 cell counting, demonstrated that GSTK1 overexpression inhibited cell proliferation, whereas GSTK1 knockdown enhanced proliferative capacity (Fig. 2A-C, J-L; Figure S2A-C, I, K). Additionally, GSTK1 overexpression increased the apoptotic rate, while GSTK1 knockdown yielded the opposite effect, the results of which were consistent with the western blot analysis (Fig. 2F, G, O, P; Figure S2F, H, N, P). Wounding assays indicated that GSTK1 overexpression shared a wider distance in HepG2 or Hep3B cells, while GSTK1 knockdown exhibited the opposite phenomenon (Fig. 2D, M; Figure S2D, L). Transwell experiments showed that GSTK1 overexpression reduced the number of cells that passed through the chamber in HepG2 or Hep3B cells, and GSTK1 knockdown increased the number of cells that passed through the chamber in HCC-LM3 or Huh7 cells (Fig. 2E, N; Figure S2E, M). These results suggested that GSTK1 inhibits HCC migration in vitro. In addition, tumor cell ATP production decreased after GSTK1 overexpression and increased following GSTK1 knockdown (Fig. 2H, Q; Figure S2G, O). The subcutaneous tumor xenograft experiment in BALB/c-Nude mice demonstrated that GSTK1 overexpression resulted in a significant reduction in tumor size and weight compared to the control (pCDH) group, whereas GSTK1 knockdown led to increased tumor volume and weight relative to the normal control (NC) group (Fig. 2I, R).

Collectively, these results demonstrate that GSTK1 inhibits HCC progression in vitro and in vivo.

### GSTK1 is responsible for L-carnitine metabolism and L-carnitine acts against HCC cells, which are enhanced by GSTK1 overexpression or weakened by GSTK1 knockdown

**Fig. 3:**
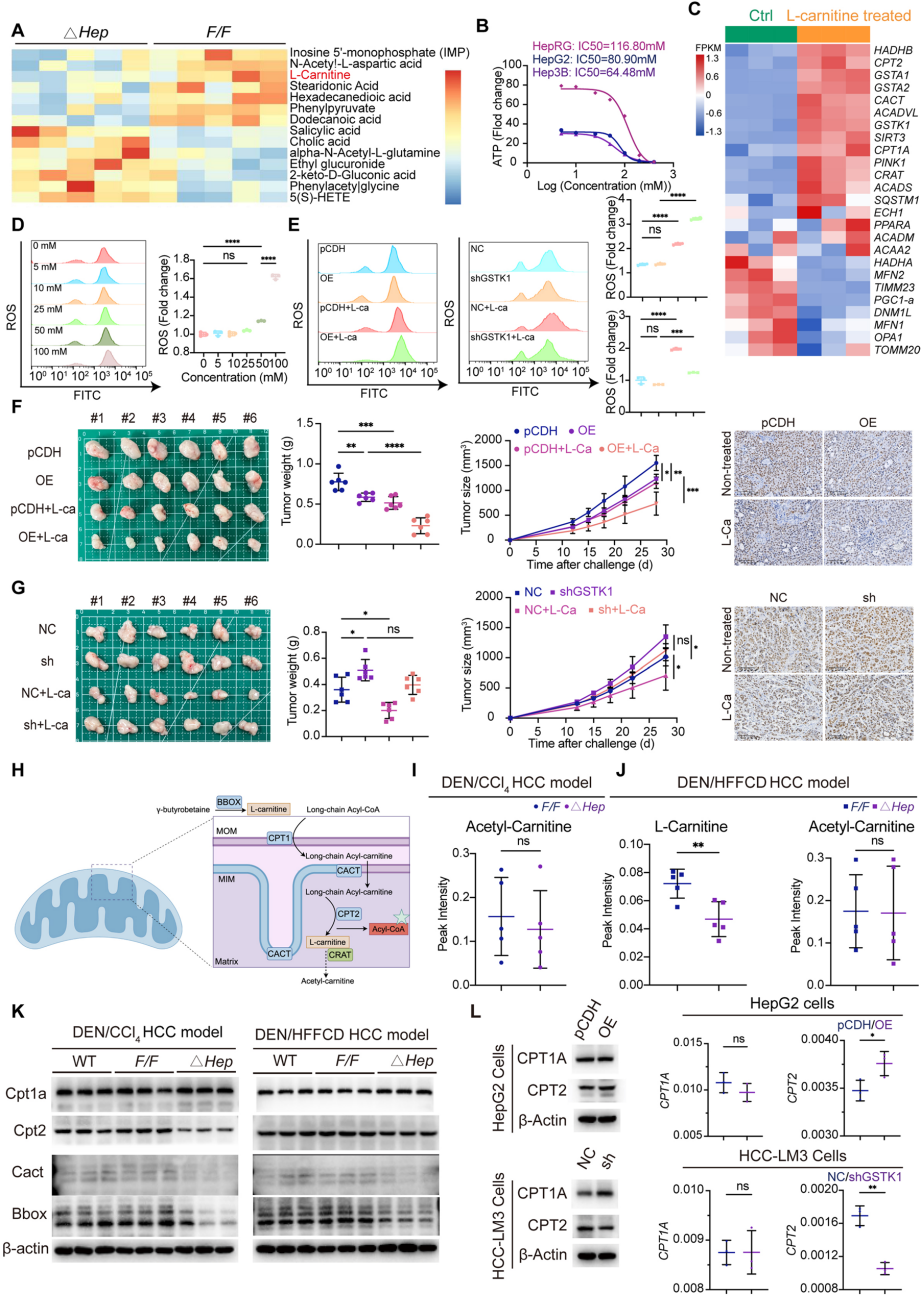
GSTK1 is responsible for L-carnitine metabolism and L-carnitine could against HCC cells and is enhanced by GSTK1 overexpression or weakened by GSTK1 knockdown. (**A**) Metabolites enriched in non-target metabolomics in DEN/CCl_4−_treated mice related to Fig. 1. (**B**) L-carnitine IC_50_ measurement in HepRG, Hep3B, and HepG2 cells detected by CellTiter-Lumi™ Plus II Luminescent Cell Viability Assay kit. (**C**) Mitochondrial fatty acid β-oxidation, mitochondrial quality control, and GSH-related genes analysis of RNA-seq with Ctrl and L-carnitine-treated HepG2 cells. (**D**) ROS detection of HepG2 cells treated with gradient concentration of L-carnitine for 48 h. (**E**) ROS detection of HepG2 cells after GSTK1 overexpression; HCC-LM3 cell GSTK1 knockdown treated with L-carnitine (100 mM) for 48 h. (**F-G**) Cells with GSTK1 overexpression or GSTK1 knockdown were transplanted on BALB/c-Nude mice, then the cells were or were not treated or with L-carnitine (*n* = 6). Immunohistochemistry staining of KI67 in sections are also shown (bar = 100 μm). (**H**) Summary schematic diagram of carnitine metabolism. (**I**) Analysis of acetyl-carnitine in DEN/CCl_4_ HCC model related to Fig. 1 and detected by ELISA. (**J**) Analysis of L-carnitine and acetyl-carnitine in DEN/HFFCD HCC model related to Fig. 1 and detected by ELISA. (**K**) Immunoblotting analysis of CPT1a, CPT2, CACT, BBOX, and β-actin in the DEN/CCl_4_ and DEN/HFFCD HCC models related to Fig. 1. (**L**) Immunoblotting and RT-qPCR analysis of CPT1A, CPT2, and β-actin in HCC cell lines related to Fig. 2. Data are presented as the mean ± SD. n.s, no significance, * *P* < 0.05, ** *P* < 0.01, *** *P* < 0.001, and **** *P* < 0.0001

RNA-sequence and non-target metabolomics analysis were employed to further explore the function of GSTK1 and elucidate the molecular mechanism underlying GSTK1 on the tumor malignant phenotype (Figure S3A-D). A low L-carnitine level was observed in *Gstk1*^*△Hep*^ mice group compared to *Gstk1*^*F/F*^ mice (Fig. 3A). Carnitine (L-carnitine) is primarily responsible for transporting long-chain fatty acids into the mitochondria for beta oxidation, thus providing energy to cells. However, studies have shown that L-carnitine has a dual role in tumor progression. Thus, the effect of L-carnitine gradient concentrations on HCC cells was determined. The number of suspended cells and the proportion of apoptotic cells were increased and analyzed by optical microscopy and flow cytometry. It is worth noting that excess L-carnitine also led to *WT* primary hepatocyte and HepRG cell death. However, the proportion of normally proliferating HepRG cells was > 90% (Figure S4A-G). IC_50_ detection of L-carnitine for HepRG, HepG2, and Hep3B cells was 116.80, 80.90, and 64.48 mM, respectively (Fig. 3B). Thus, 100 mM was used for subsequent experiments.

RNA-seq was performed to compare control and L-carnitine-treated HepG2 cells in order to elucidate the molecular mechanisms underlying L-carnitine-induced cytotoxicity in HCC cells. Differential gene analysis showed that *GSTA1*, *GSTA2*,* PGC*, *STAR*, and *CHKA* are most significantly regulated and KEGG results showed that metabolic pathways were most significantly regulated (Figure S3E, F). Mitochondrial fatty acid β-oxidation-related genes, such as *CPT1A* and *CPT2*, were upregulated and MQC-related genes were all downregulated **(**Fig. 3C; Figure S3G). ROS production was examined after gradient L-carnitine stimulation of HCC cells. L-carnitine stimulated by gradient concentration promoted ROS production and immunoblotting analysis showed inhibition of mitochondrial biosynthesis, mitochondrial fusion and fission, and mitophagy (Fig. 3D; Figure S3H). These findings suggest that L-carnitine exerts cytotoxic effects on HCC cells by inducing oxidative stress and triggering mitochondrial dysfunction.

The CCK8 assay, ROS detection, and flow cytometry results showed that overexpression of GSTK1 enhances the function of L-carnitine against HCC, while GSTK1 knockdown yielded the opposite effect (Fig. 3E; Figure S4H-J). Cells stably overexpressing GSTK1 or GSTK1 knockdown were subcutaneously injected into BALB/c-Nude mice and fed with L-carnitine in water (4 mg/ml) every day for 28 d to assess the impact of L-carnitine on HCC in vivo. Tumors in the L-carnitine-treated group exhibited a significant reduction in size and weight compared to the pCDH/NC group. Notably, the decrease between the GSTK1 overexpression (OE) group and OE + L-carnitine groups was larger than the pCDH group compared to pCDH + L-carnitine group, while there was no significant difference following GSTK1 knockdown. Ki67 labelling in sections from the four groups shared the same trend (Fig. 3 F, G). These results indicate that high expression of GSTK1 promoted the killing ability of L-carnitine against HCC.

### GSTK1 regulates key transporters of L-carnitine metabolism through MQC

L-carnitine metabolism is achieved through the CPTS, which mainly consists of CPT1, CPT2, and CACT. The endogenous synthesis of L-carnitine depends on gamma-butyrobetaine hydroxylase (BBOX; Fig. 3H) [17]. Non-target metabolomics analysis (Fig. 3A) also indicated that acetyl-carnitine was unchanged between the *Gstk1*^*△Hep*^ and *Gstk1*^*F/F*^ groups, as detected in the DEN/HFFCD murine model (Fig. 3I-J).

The protein and mRNA levels of genes related to L-carnitine metabolism further clarified how GSTK1 affects L-carnitine metabolism. Immunoblotting and RT-qPCR analysis revealed that CPT1A was increased and CPT2, CACT, and BBOX decreased in *Gstk1*^*△Hep*^ mice, while immunohistochemistry had the same results (Fig. 3K; Figure S5A-C). The above results in HCC cell lines with GSTK1 overexpression or knockdown were verified in vitro. GSTK1 expression was highly consistent with CPT2 expression, i.e., high-expression GSTK1 had higher CPT2 expression. CPT1A and CACT expression may be heterogeneous according to cell type (Fig. 3L; Figure S5D-G).

**Fig. 4:**
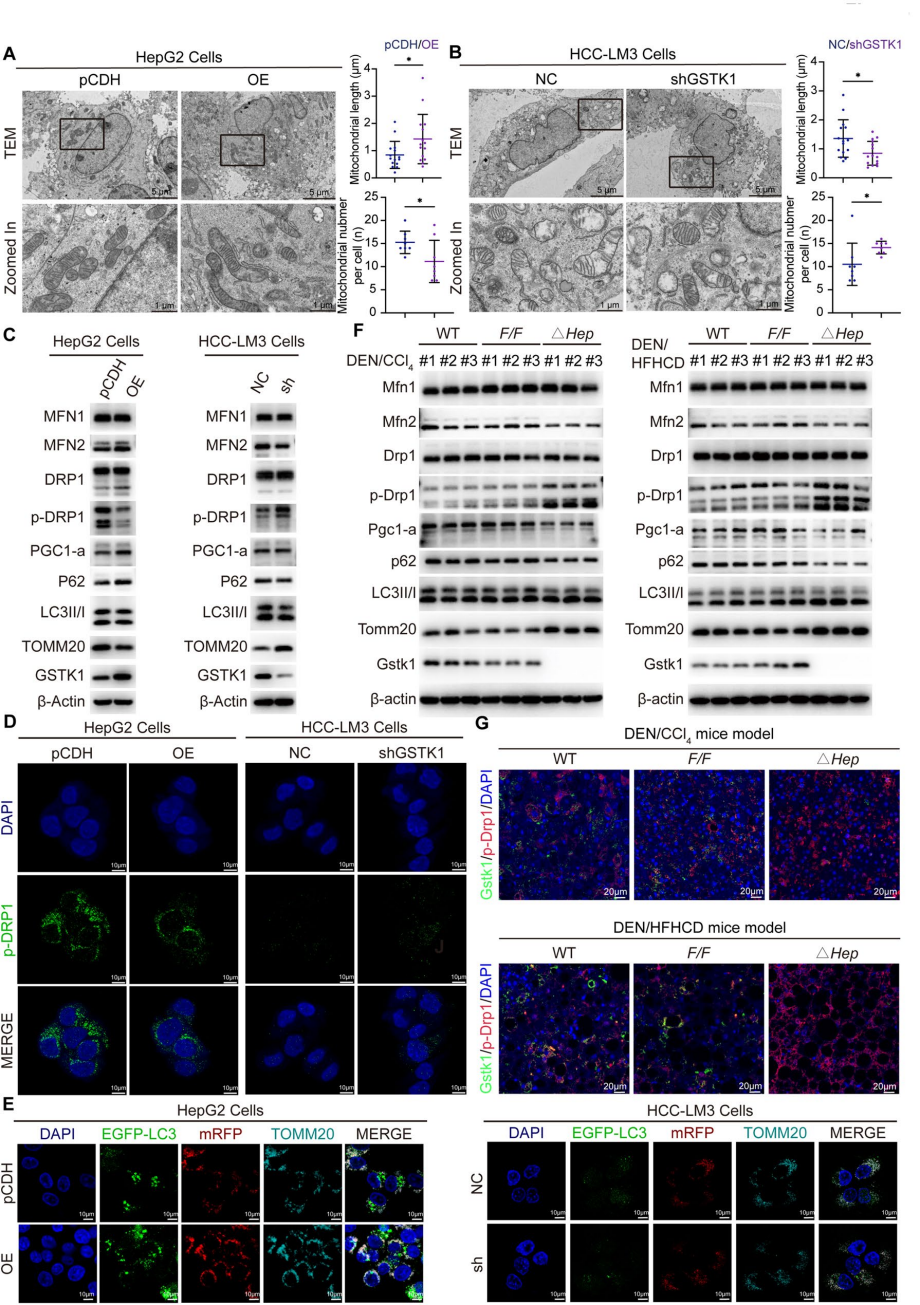
GSTK1 promotes mitochondrial biosynthesis and mitochondrial fusion, while inhibiting mitochondrial fission and mitophagy. (**A-B**) Representative TEM images of mitochondria in HCC cells lines related to Fig. 2 (bar = 5–1 μm). (**C**) Immunoblotting analysis of MFN1, MFN2, DRP1, p-DRP1, PGC1-α, P62, LC3II: I ratio, TOMM20, GSTK1, and β-actin in HCC cells lines related to Fig. 2. (**D**) Immunofluorescent staining of p-DRP1(S616) in HCC cells lines related to Fig. 2 (bar = 10 μm). (**E**) Immunofluorescent staining of TOMM20 in HepG2 and HCC-LM3 cells stably transfected (mRFP-EGFP-LC3B), then transiently transfected (OE-GSTK1 or shGSTK1 [bar = 10 μm]). (**F**) Immunoblotting analysis of Mfn1, Mfn2, Drp1, p-Drp1, Pgc1-α, p62, LC3II/I, Tomm20, GSTK1, and β-actin in mice tumor tissues related to Fig. 1. (**G**) Immunofluorescent staining of GSTK1 and p-Drp1(S616) in liver sections from DEN/CCl_4_- or DEN/HFFCD-treated various mice related to Fig. 1 (bar = 20 μm). n.s, no significance, * *P* < 0.05

**Fig. 5:**
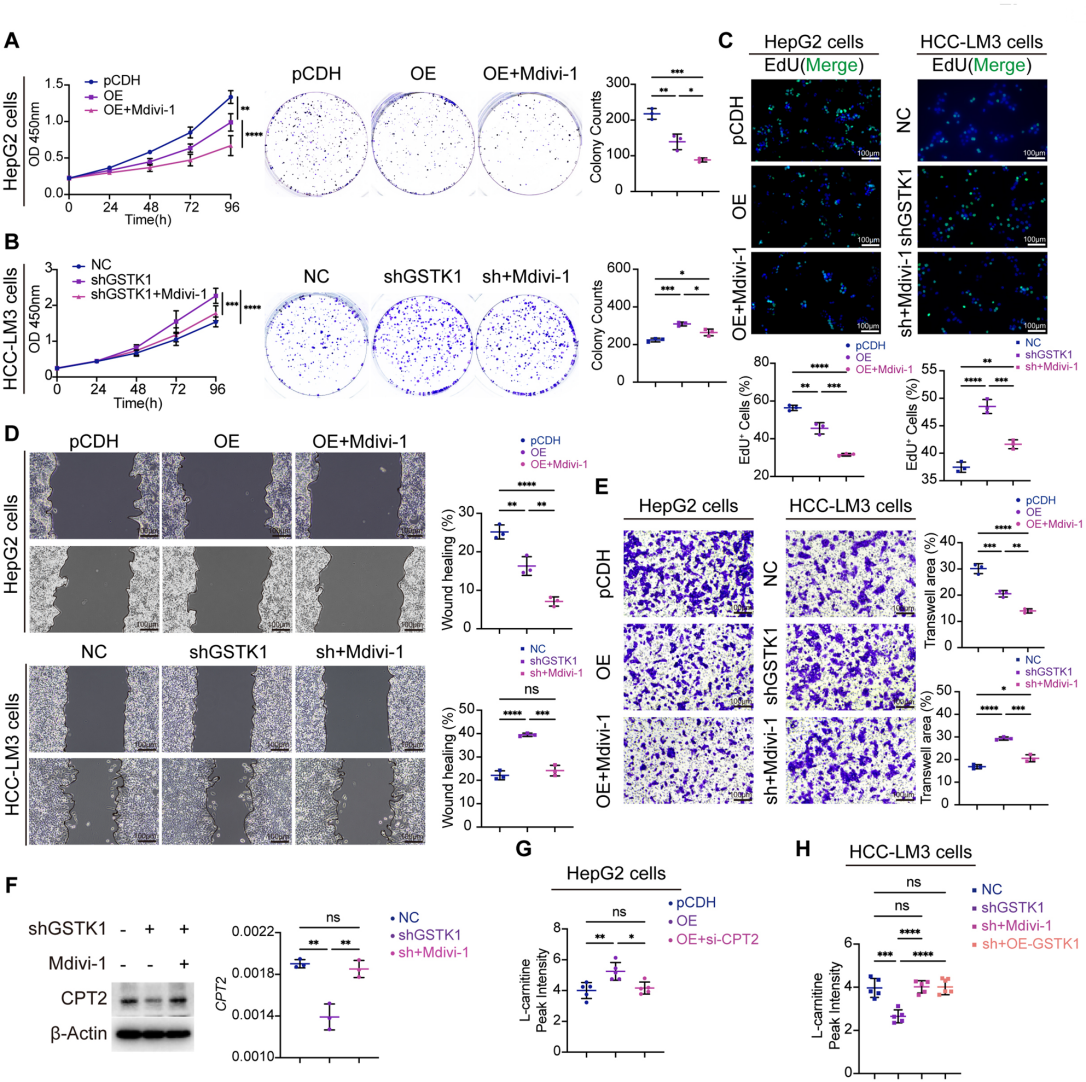
Mdivi-1 restores GSTK1 function on HCC cells and CPT2 levels. (**A-C**) Cell proliferation ability of HCC cells lines (Fig. 2) and treated with Mdivi-1. CCK8, colony formation (**A-B**), EdU (**C**) bar = 100 μm. (**D-E**) Cell migration ability of HCC cells lines related to Fig. 2 and treated with Mdivi-1. Wound healing (**D**), Transwell (**E**) bar = 100 μm. (**F**) Immunoblotting and RT-qPCR analysis of CPT2 in HCC-LM3 cells with or without GSTK1 knockdown and Mdivi-1. (**G**) Analysis of L-carnitine in HepG2 cells and GSTK1 overexpression with or without si-*CPT2* detected by ELISA. (**H**) Analysis of L-carnitine in HCC-LM3 cells and GSTK1 knockdown with or without Mdivi-1 or restored GSTK1 detected by ELISA. Data are presented as the mean ± SD. n.s, no significance, * *P* < 0.05, ** *P* < 0.01, *** *P* < 0.001, and ****P < 0.0001

Because L-carnitine metabolism mainly occurs in mitochondria, a transmission electron microscope was used to capture images of mitochondria. Mitochondrial length was longer in GSTK1 overexpression cells and was shorter in GSTK1 knockdown cells. The number of mitochondria was reduced in GSTK1 overexpression cells and increased in GSTK1 knockdown cells, which suggested changes in the expression of MQC-related proteins (Fig. 4A, B; Figure S6A, B). PGC1-a, MFN2, and P62 were increased, and TOMM20, p-DRP1(Ser616), the LC3II: I ratio were decreased in cells overexpressing GSTK1. PGC1-a, MFN2, and P62 were decreased, and TOMM20, p-DRP1(Ser616), and the LC3II: I ratio were increased in GSTK1 knockdown cells. MFN1 and DPR1 were unchanged, as analyzed by immunoblotting (Fig. 4C; Figure S6C-J). Similar results were obtained by immunofluorescent staining (Fig. 4D, E). In addition, increased Tomm20, p-Drp1(Ser616), and the LC3II: I ratio were observed in DEN/CCl_4_- and DEN/HFFCD-treated *Gstk1*^*△Hep*^ mice with decreased Pgc1-a, Mfn2, and p62, as analyzed by immunoblotting (Fig. 4F). Multi-immunofluorescent staining showed that p-Drp1(Ser616) fluorescence intensity was higher in the *Gstk1*^*△Hep*^ mice group (Fig. 4G). Thus, these results demonstrated that GSTK1 regulate the MQC, particularly by promoting mitochondrial biosynthesis and mitochondrial fusion, while inhibiting mitochondrial fission and mitophagy. Therefore, we speculated whether GSTK1 affects the MQC and thus regulates CPT2. Mdivi-1 was used to block mitochondrial fission. It was found that CPT2 expression could be restored in GSTK1 knockdown cell lines if Mdivi-1 was used simultaneously (Fig. 5F). L-carnitine levels were shown to be decreased in overexpressed GSTK1 cells with si-CPT2. Restoring GSTK1 expression or using Mdivi-1 in GSTK1 knockdown cells increased L-carnitine levels (Fig. 5G, H). In addition, blocking mitochondrial fission inhibited GSTK1 function on HCC cells. Use of Mdivi-1 decreased the proliferation and migration of GSTK1 knockdown on HCC cells (Fig. 5A-E).

Taken together, these results suggest that GSTK1 is involved in L-carnitine metabolism and affects the key L-carnitine regulatory enzymes. The ability of L-carnitine to kill HCC is enhanced by high GSTK1 expression. Therefore, use of L-carnitine could be a strategy for HCC treatment.

### GSTK1 competes with DRP1 for binding to PGAM5

**Fig. 6:**
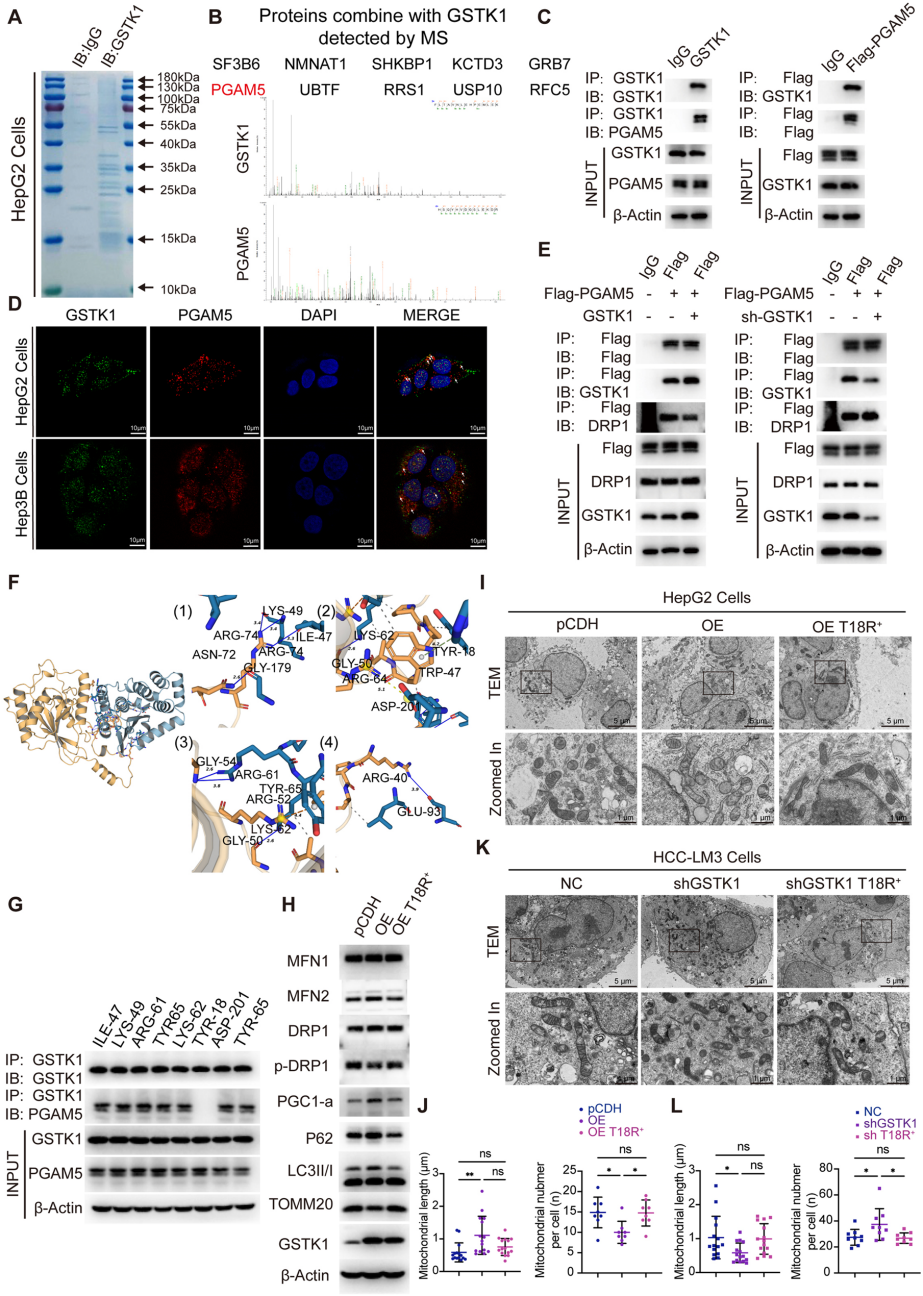
GSTK1 competes with DRP1 for binding to PGAM5. (**A-B**) Mass spectrometry (MS) analysis of GSTK1-associated proteins. Total cell extracts of HepG2 cells stably overexpressing GSTK1 was subjected to affinity purification with anti-GSTK1 antibody. The purified protein complex was resolved on SDS-PAGE and Coomassie brilliant blue staining, then the bands were excised and analyzed by mass spectrometry. (**C**) Immunoprecipitation and immunoblotting analysis of the GSTK1 and PGAM5 interaction in HepG2 cells overexpressing GSTK1 or stably transfected Flag-PGAM5. (**D**) Immunofluorescent staining of the GSTK1 and PGAM5 interaction in HepG2 and Hep3B cells (bar = 10 μm). (**E**) Immunoprecipitation and immunoblotting analysis of the PGAM5 and DRP1 interaction in HepG2 cells overexpressing GSTK1 or transiently transfected knockdown GSTK1. (**F**) The prediction of specific binding sites for the GSTK1 and PGAM5 interaction detected by PLIP software. (**G**) Immunoprecipitation and immunoblotting analysis of the GSTK1 and PGAM5 interaction in HepG2 cells transiently transfected point mutation plasmids. (**H**) Immunoblotting analysis of MFN1, MFN2, DRP1, p-DRP1, PGC1-α, P62, the LC3II: I ratio, TOMM20, GSTK1, and β-actin in HepG2 cells overexpressing GSTK1 and a TYR-18 point mutation. (**I-J**) Representative TEM images of HepG2 cells overexpressing GSTK1 and a TYR-18 point mutation (bar = 5–1 μm). (**K-L**) Representative TEM images of HCC-LM3 cells and GSTK1 knockdown and a TYR-18 point mutation (bar = 5–1 μm). n.s, no significance, * *P* < 0.05, ** *P* < 0.01

To understand how GSTK1 regulates the MQC, GSTK1-associated proteins were isolated from HepG2 cells by immunoaffinity purification and analyzed by liquid chromatography and high-throughput mass spectrometry (LC-MS/MS; Fig. 6A). None of the MQC proteins were directly detected, suggesting that GSTK1 did not directly interact with MQC. However, the protein, PGAM5, was shown to interact with DRP1. PGAM5 recruits DRP1 and activates DRP1 GTPase activity by dephosphorylating the serine 637 site of DRP1 upon induction of necrosis [18]. The interaction between endogenous GSTK1 and PGAM5 proteins was confirmed by co-immunoprecipitation in combination with immunoblotting analysis or immunofluorescent staining (Fig. 6B-D; Figure S7A).

Whether or not GSTK1 affects the PGAM5 and DRP1 interaction was determined. GSTK1 overexpression led to reduced binding of PGAM5 and DRP1, while GSTK1 knockdown increased binding of PGAM5 and DRP1 (Fig. 6E; Figure S7B). In addition, PLIP was used to predict the specific binding sites of GSTK1 and PGAM5. Interaction analysis was performed using GSTK1 as the reference chain, with gold representing PGAM5 and blue representing GSTK1 (Fig. 6F). Hydrogen bonds formed between GSTK1 LYS-62 and PGAM5 GLY-50 (blue solid line), Pi-Pi stacking interaction formed between GSTK1 TYR-18 and PGAM5 TRP-47 (green dashed line), and a salt bridge formed between GSTK1 GLU-201 and PGAM5 GLY-50 (yellow dashed line). A Pi cation interaction formed between GSTK1 TYR-65 and PGAM5 ARG-52 (orange dashed line). In addition, there were multiple sets of hydrophobic interactions (gray dashed line; Fig. 6F; Figure S7C, D). Cell lines with different point mutation sites were then constructed according to the PLIP results. Co-immunoprecipitation and immunoblotting analysis showed that TYR-18 was essential for combining GSTK1 and PGAM5, and the TYR-18 mutation restored the regulatory effect of GSTK1 on the MQC protein, which was consistent with the transmission electron microscopy results (Fig. 6G-L; Figure S7E).

In summary, these results demonstrate that GSTK1 prevents the association between PGAM5 and DRP1 and was dependent on the TYR-18 domain, thereby halting mitochondrial fission and mitophagy.

### GSTK1 is regulated by peroxisome proliferator activated receptor alpha (PPARα)/retinoid x receptor alpha (RXRα) and the RXRα agonist, bexarotene, inhibited the proliferation and migration of tumor cells

**Fig. 7:**
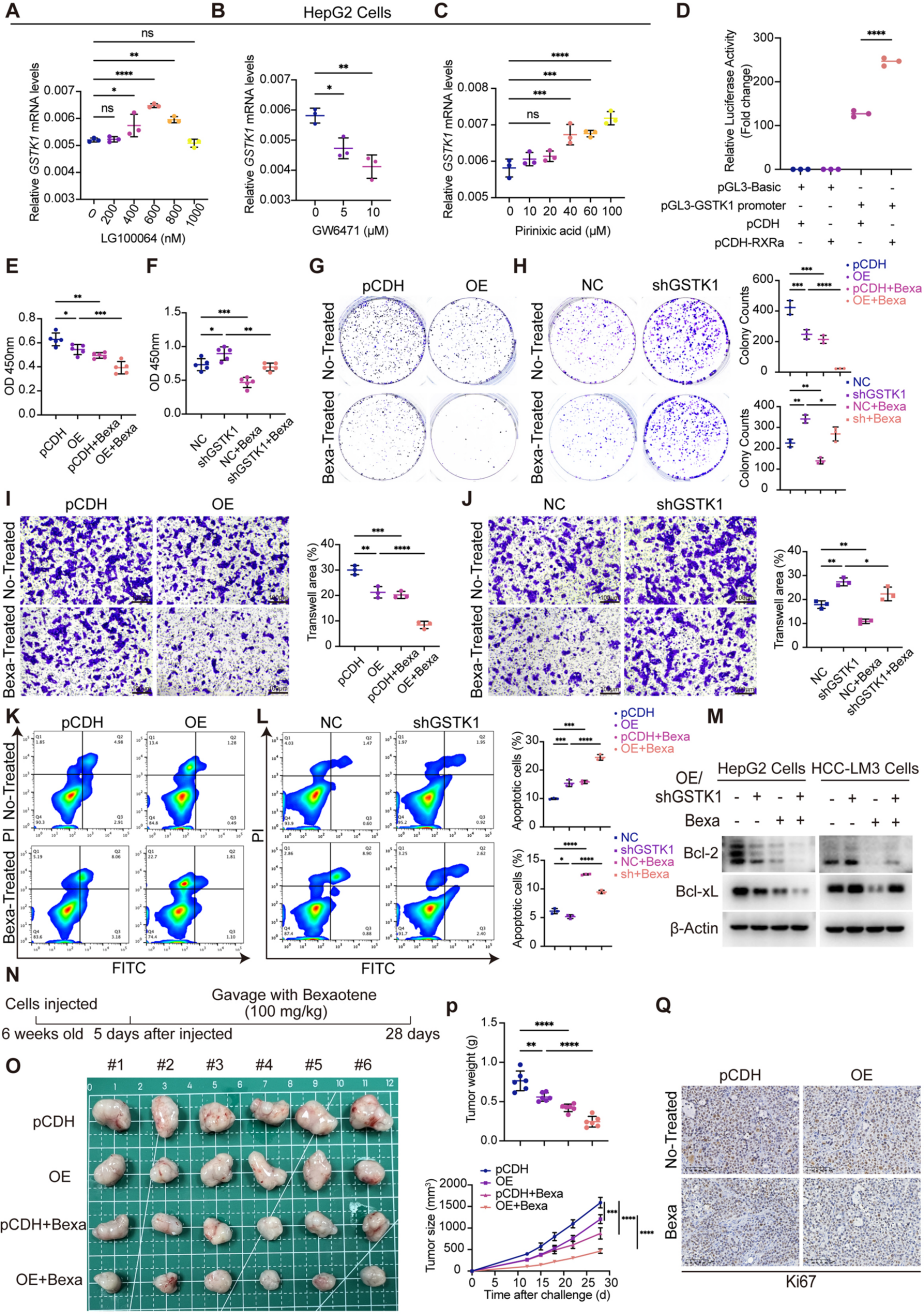
GSTK1 is regulated by PPARα/RXRα and Bexarotene could inhibit the proliferation and migration of tumor cells, enhanced by GSTK1 overexpression or weaken by GSTK1 knockdown (**A-C**) RT-qPCR analysis of *GSTK1* mRNA levels in HepG2 cells treated with LG100064 (**A**), GW6471 (**B**), Pirinixic acid (**C**). (**D**) The luciferase reporter gene assay of RXRα and GSTK1. (**E-H**) Cell proliferation ability detection of HepG2 cells overexpress GSTK1 or HCC-LM3 cells knockdown GSTK1 treated with Bexarotene (40 µM), detected by CCK8 (**E-F**), Colony formation (**G-H**). (**I-J**) Cell migration ability detection of HepG2 cells overexpress GSTK1 or HCC-LM3 cells knockdown GSTK1 treated with Bexarotene (40 µM), detected by Transwell (Bar = 100 μm). (**K-L**) The apoptosis ratio of HepG2 cells overexpress GSTK1 or HCC-LM3 cells knockdown GSTK1 treated with Bexarotene (40 µM), detected by flow cytometry using the Annexin V-FITC/PI staining kit. (**M**) Immunoblotting analysis of Bcl-xL, Bcl-2, and β-Actin in HepG2 cells overexpressing GSTK1 or HCC-LM3 cells with GSTK1 knockdown following treatment with Bexarotene (40 µM). (**N-O**) Hep3B cells with GSTK1 overexpression were transplanted on BALB/c-Nude mice, then treated or not treated with Bexarotene, and tumor volumes and tumor weights were recorded (*n* = 6). Schematic diagram of Bexarotene treated model (**N**). (**Q**) Immunohistochemistry staining of Ki67 (Bar = 100 μm). Data are presented as mean ± SD. n.s, no significance, * *p* < 0.05, ** *p* < 0.01, *** *p* < 0.001 and **** *p* < 0.0001

The PROMO database was used to forecast upstream GSTK1 transcription factors; PPARα/RXRα was of particular interest (Figure S7F). To verify this conclusion, an RXRα agonist (LG100064), a PPARα inhibitor (GW6471), and a PPARα agonist (pirinixic acid) were used. The PPARα/RXRα agonist increased GSTK1 expression, while the inhibitor decreased GSTK1 expression (Fig. 7A-C). Importantly, the luciferase reporter gene assay confirmed that RXRα was an upstream promoter of GSTK1 (Fig. 7D). RT-qPCR and immunoblotting analysis showed that siRNA for RXRα also downregulated GSTK1 (Figure S7G). RXRα, an important transcription factor, was also shown to be involved in the progression of various tumors, such us acute myeloid leukemia and prostate cancer [19]. However, no studies have specifically verified the role of RXRα in HCC. Bexarotene is a high-affinity and selective retinoid X receptor (RXR) agonist that has been used for research involving cutaneous T-cell lymphomas. Clinical trials related to breast and lung cancer have also been carried out [20, 21]. Herein the function of bexarotene in HCC cells was determined. Cell proliferation ability was detected by CCK8 cell counting and the colony formation assay, which showed that HepG2 or HCC-LM3 cell proliferation was significantly inhibited following bexarotene treatment, then strengthened by GSTK1 overexpression or weakened by GSTK1 knockdown (Fig. 7E-H). The Transwell assay confirmed that bexarotene inhibited cell migration and high GSTK1 expression enhanced this phenomenon (Fig. 7I, J). In addition, flow cytometry provided more intuitive results. Bexarotene promoted apoptosis of tumor cells, while GSTK1 overexpression further increased the apoptosis ratio. Immunoblotting analysis obtained the same results (Fig. 7K-M). Hep3B cells stably overexpressing GSTK1 were subcutaneously injected into BALB/c-Nude mice to assess the impact of bexarotene on HCC in vivo, then subsequently gavaged with bexarotene (100 mg/kg) every day (Fig. 7N). Tumors in the OE group and both bexarotene-treated groups exhibited a significant reduction in size and weight compared to the pCDH group. Notably, the decrease between the OE and OE + bexarotene groups was greater than the pCDH group compared to the pCDH + bexarotene group. Ki67 labelling in sections from the four groups of mice exhibited the same trend (Fig. 7O-Q).

In general, these findings demonstrate that GSTK1 is regulated by PPARα/RXRα and bexarotene inhibited the proliferation and migration of HCC cells in vitro and in vivo, which was also enhanced by high GSTK1 expression.

### GSTK1 expression is decreased in HCC tissues and related to patient recurrence and prognosis

**Fig. 8:**
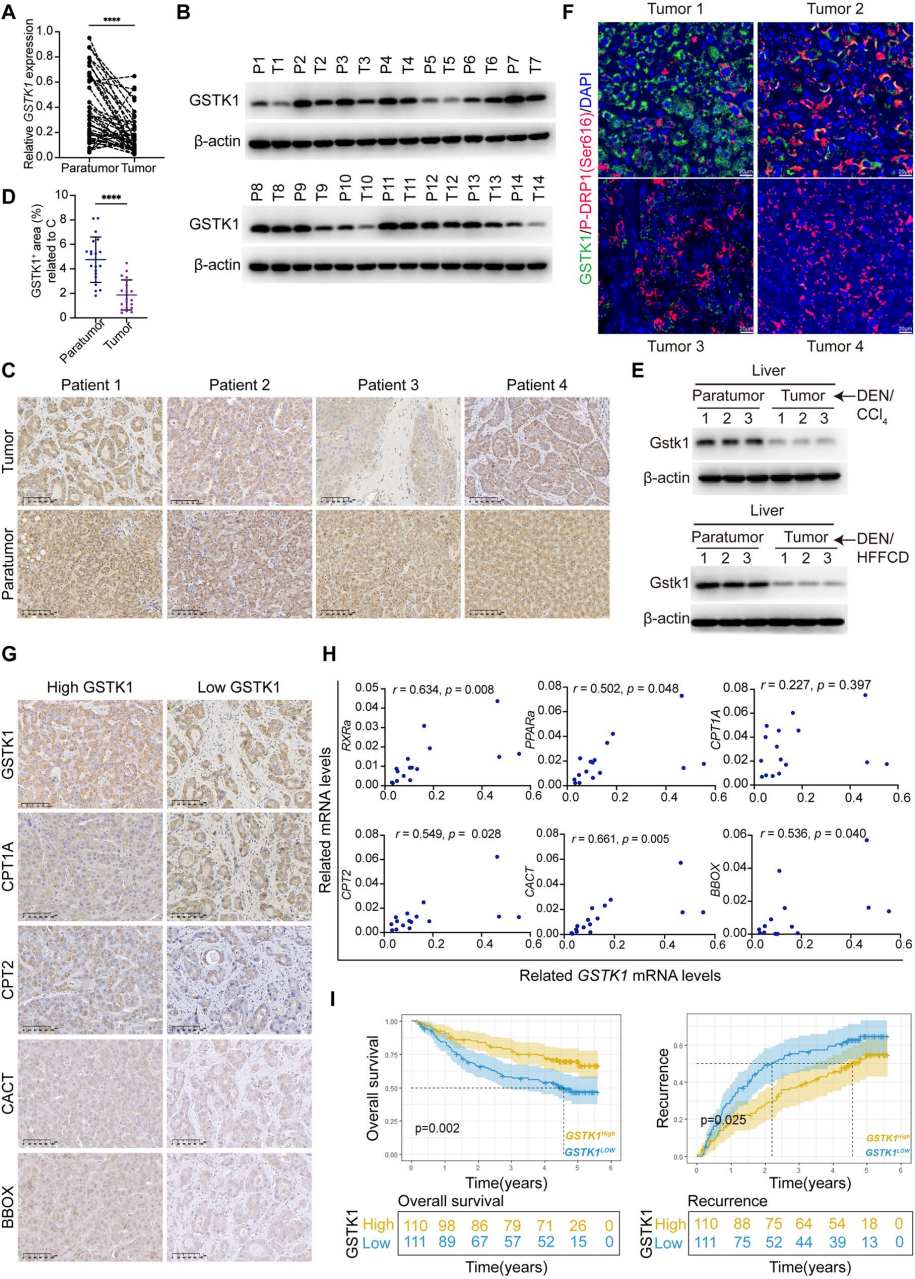
Low GSTK1 corelates with poor prognosis and high recurrence of human HCCs. (**A-B**) GSTK1 expression in paired human HCC and para-tumor tissues (*n* = 40) detected by RT-qPCR (**A**) and immunoblotting analysis (*n* = 14; **B**) (**C-D**) Immunohistochemistry staining of GSTK1 in human HCC and para-tumor tissue sections (bar = 100 μm). (**E**) Immunofluorescent staining of GSTK1 and p-DRP1(Ser616) in human HCC sections (bar = 20 μm). (**F**) Immunoblotting analysis of GSTK1 in liver tumors and paired para-tumor tissues of DEN/CCl_4_- or DEN/HFFCD-treated *WT* mice (*n* = 3). (**G**) Immunohistochemistry staining of GSTK1, CPT1A, CPT2, CACT, and BBOX in human HCC sections (bar = 100 μm). (**H**) Correlation analysis of mRNA levels between *GSTK1*, *PPARα*, *RXRα*, *CPT1A*, *CPT2*, *CACT*, and *BBOX* in human HCCs detected by RT-qPCR (*n* = 16). (**I**) Correlation between GSTK1 expression with patient overall survival and recurrence (GSE14520). Data are presented as the mean ± SD. n.s, no significance, * *P* < 0.05, ** *P* < 0.01, *** *P* < 0.001, and **** *P* < 0.0001

GSTK1 expression in pairs of tumor and para-tumor tissues from HCC patients was examined using RT-qPCR, western blot analysis, and IHC to determine the clinical relevance of GSTK1 in HCC. Apparently, the levels of GSTK1 mRNA and protein were decreased in tumors compared to para-tumor tissues (Fig. 8A-D). GSTK1 was degraded in liver tumors in DEN/CCl_4_- and DEN/HFFCD-induced HCC related to Fig. 1 (Fig. 8E). IF and IHC staining showed that lower GSTK1 expression in tumor tissues had higher expression of p-DRP1 (Ser616) or lower CPT2, CACT, and BBOX (Fig. 8F, G). Thus, like DEN/CCl_4_- and DEN/HFFCD-treated *Gstk1*^*△Hep*^ mice, the samples with reduced GSTK1 expression displayed an obvious mitochondrial fission and poor L-carnitine metabolism phenotype (Figs. 3 and 6).

The GEPIA2 database was used to further confirm the relationship between GSTK1 and the MQC and L-carnitine metabolism genes. *GSTK1* was positively correlated with *MFN2*, *MAP1LC3A*, *PGC1-*α, *PPAR*α, *RXR*α, *CPT2*, *CACT*, and *BBOX*, negatively correlated with *DRP1* and *TOMM20*, and not significantly correlated with *MFN1*, *P62*, and *MAP1LC3B*. The *GSTK1* mRNA level was positively connected to *PPAR*α, *RXR*α, *CPT2*, *CACT*, and *BBOX* mRNA levels but not related to CPT1A (Fig. 8H; Figure S8).

Online public databases (GSE14520 and HCCDB8) were analyzed to determine the prognostic value of GSTK1 in human HCC. Patients with low GSTK1 expression were more likely to have poor overall survival [OS] (*P* = 0.002) and early tumor recurrence (*P* = 0.025; Fig. 8I). Low GSTK1 expression was associated with a high risk of metastasis, advanced TNM/BCLC/CLIP staging, and elevated AFP levels (Table S1 and Figure S8O).

## Discussion

HCC typically has unfavorable clinical outcomes and is the only major cancer for which death rates have not improved over the last 10 years. Given the limited efficacy of existing therapies, there is a pressing need to identify novel molecular targets to enhance the prognosis of HCC. In the current investigation, a significant correlation was observed between GSTK1 expression and overall survival (OS) in HCC patients, as well as clinicopathological features. Mechanistically, hepatocyte *Gstk1* loss promoted HCC progression by regulating L-carnitine metabolism and MQC.

Cancer metabolism is a complex network with high plasticity. L-carnitine may enhance fatty acid oxidation, which contributes to this plasticity, thereby furnishing tumor cells with additional energy sources, which in turn supports tumor growth and proliferation, and inhibits tumor cell apoptosis. However, this view is not consistent with clinical observations. Patients with low L-carnitine expression may have a higher recurrence rate and poorer survival [22]. Moreover, cancer patients are often accompanied by energy metabolism disorders. Indeed, L-carnitine supplementation may improve the nutritional status and quality of life of cancer patients [23]. The current study, for the first time, demonstrated that L-carnitine suppresses HCC cell proliferation in vivo and in vitro. Mechanistically, L-carnitine exerted an anti-tumor effect by promoting ROS production, which subsequently caused mitochondrial dysfunction. Notably, GSTK1 overexpression enhanced L-carnitine killing ability of HCC cell lines. GSTK1 overexpression inhibited mitochondrial fission and mitophagy. Therefore, clinical strategies involving L-carnitine for HCC may include targeting key acting enzymes (e.g., GSTK1, CPT1A, and CPT2), combining L-carnitine with other drugs (e.g., Mdivi-1 and mitophagy inhibitors), and implementing nutritional interventions.

GSTK1 was shown to participate in regulating MQC, specifically by promoting mitochondrial biosynthesis and mitochondrial fusion, while inhibiting mitochondrial fission and mitophagy. Consistent with previous research [24], GSTK1 downregulation amplified the gene and protein expression of mitochondrial fission factor via the JNK pathway, enhancing the ability to recruit DRP1 to mitochondria. However, the current study innovatively demonstrated the mechanism by which GSTK1 functions through binding to PGAM5, competing with DRP1 and dependent on the TYR-18 site. A growing body of research has shown that MQC is involved in tumor progression and specifically on how mitochondrial dynamics act directly on tumor cells or indirectly on cells responsible for tumor aggression and defense [24]. Enhanced DRP1 protein and mRNA expression and shorter mitochondrial size were detected in HCC tumors, suggesting enhanced mitochondrial fission in tumors. Overexpression of DRP1 in HCC cells has been linked to enhanced tumor growth in vivo and vice versa, and DRP1 deficiency causes reduced tumor growth. Interestingly, DRP1 expression is strongly increased in distant HCC metastases compared to primary HCC [25]. Mitochondrial fission induces glycolytic reprogramming in cancer-associated-fibroblasts, which drives stromal lactate production and tumor growth. Tumor-infiltrating T lymphocytes commonly undergo exhaustion during cancer progression. PD-1 signaling inhibits mitochondrial fragmentation in T cells by downregulating Drp1 phosphorylation on Ser616, likely through regulation of the ERK1/2 and mTOR pathways. Tumor-associated-macrophage recruitment and polarization is facilitated by mitochondrial fission, which causes cytosolic mtDNA stress and increases CCL2 secretion by cancer cells [26]. Mitochondrial dynamics may thereby represent an attractive therapeutic target for various cancers. Understanding the molecular mechanism underlying the regulation of mitochondrial dynamics is important for developing therapies against cancer. The current study showed that low GSTK1 expression is associated with high p-DRP1 (Ser616) expression and further promoted the proliferation of HCC.

Finally, RXRs are vital in transcription. RXRs have been reported to be involved in diverse cellular networks from cell proliferation to lipid metabolism and are critical for normal eye development [27]. Apart from controlling normal physiology, RXRs also regulate cancer-associated virus infections. While RXR agonists impair HBV infection in liver cancer cell lines and primary hepatocytes, reduced RXRα expression increases HBV infectivity [28]. The results herein showed that the RXRα agonist, bexarotene, inhibited HCC cells proliferation in vitro and in vivo; research involving RXRα in HCC warrants further research.

The current study had several limitations. First, the effects of L-carnitine were not determined in knockout mice, but only in a subcutaneous tumor model. Second, the effect of blocking PGAM5 on GSTK1 regulation of HCC was not elucidated.

The current study revealed that GSTK1 regulates L-carnitine metabolism and MQC via the PGAM5/DRP1 complex. In addition, GSTK1 was activated by PPARα/RXRα activator and RXRα was the GSTK1 upstream promoter. The current study illustrated that GSTK1 has a crucial role in HCC development and the RXRα/GSTK1/L-carnitine axis presents a potentially effective therapeutic strategy for HCC.

## Methods and materials

### Murine liver cancer models

*Gstk1*^*Flox/Flox*^ (also named *DsbA-L*^*Flox/Flox*^, *Gstk1*^*F/F*^) mice were gifted from Professor Feng Liu. To generate mice with hepatocyte-specific knockout of *Gstk1* (*Gstk1*^*△hep*^), Albcre/cre mice were crossed with *Gstk1*^*F/F*^ mice. C57BL/6 mice (RRID: IMSR_JAX: 000664) were purchased from GemPharmatech. 6–8-week-old male and female mice were used for all experiments. Mice were housed in filter-topped cages on autoclaved food and water. Chemically-induced HCC was achieved in different groups of mice (WT, *Gstk1*^*F/F*^, and *Gstk1*^*△hep*^) with N-nitrosodiethylamine (DEN [25 mg/kg i.p.]) at 2 weeks postpartum followed by weekly injections of carbon tetrachloride (CCl_4_ [0.5 mL/kg i.p. dissolved in corn oil]). Mice were sacrificed when 5 months old for further studies. MASH-HCC was induced in male mice with DEN (25 mg/kg i.p. [Sigma-Aldrich, N0258]) at 2 weeks postpartum and fed a high-fat, high-fructose, and high-cholesterol diet (HFFCD) at 4 weeks of life, which contained 40% calories (kcal%) from fat (mostly palm oil), 20 kcal% from fructose, and 2% cholesterol (Research Diet, D09100310). Mice were sacrificed when 6 months later. The experiments were approved by the Animal Ethics Committee of The Affiliated Drum Tower Hospital of Nanjing University Medical School and the Animal Ethics Committee of Anhui medical university. The approval number is ICAUC-2,502,011.

### Clinical sample acquisition

Paired HCC and para-tumor tissues were collected from the Affiliated Drum Tower Hospital of Nanjing University Medical School. Each patient provided informed consent for tissue analysis prior to surgery, which was approved by the institutional ethics committee of the Affiliated Drum Tower Hospital of Nanjing University Medical School. The approval number is 2018-099-02.

### Cell lines and cell culture

Hepatoblastoma-derived cell lines (HepG2 [RRID: CVCL_0027] and HCC cell lines Hep3B [RRID: CVCL_0326], SMMC-7721 [RRID: CVCL_0534], HCC-LM3 [RRID: CVCL_6832], Huh7 [RRID: CVCL_B7TI]) purchased from the Shanghai Institute for Biological Science (China) and normal control hepatocyte cell line (HepRG) purchased from ThermoFisher Scientific (USA) were cultured in DMEM supplemented with 10% FBS (WISENT, 086–150), 100 U/mL penicillin, and 100 mg/mL streptomycin (WISENT, 450-201-EL) at 37℃ in a humidified incubator with 5% CO_2_.

### Isolation of primary liver cells

The liver was successively perfused with Hank’s solution without Ca^2+^ and Mg^2+^ (Gibco, 14175095) supplemented with EGTA (MERCK, 324628), and Hank’s solution containing Ca^2+^ and Mg^2+^ (Gibco, 14025076) supplemented with collagenase IV (MERCK, 17104019). After perfusion, the liver was removed, placed in complete DMEM and minced with sterile tweezers. The homogenate was filtered through a 70 μm strainer and then centrifuged three times at 50×g for 3 min at 4℃. 45% Percoll (MERCK, P4937) solution was then added to the precipitation. The primary liver cells were acquired after centrifugation at 250×g for 5 min.

### Cell transfection

#### Stable transfection

Lentiviruses stably overexpressing GSTK1/shGSTK1 were transfected into HCC cells seeded in six-well plates, followed by treatment with polybrene (10 µg/ml, MedChenExpress, HY-112735) for 24 h.

#### Transient transfection

siRNAs were used for transient transfection. Transfection reagents, siRNA, and Opti-MEM (ThermoFisher Scientific, 31985070) were mixed according to the manual, then added to the cells, after being left at room temperature for 20 min.

### Cell stimulation experiment

Gradient concentration of L-carnitine (MedChenExpress, HY-B0399) was added to the complete culture medium for 24–48 h to determine the final stimulation concentration (0, 5, 10, 25, 50, 100, 200, 400 mM).

For Bexarotene (MedChenExpress, HY-14171), 40 µM concentration was used according to the previous study.

To explore the upstream promoter mechanism of GSTK1, *RXRa* agonist LG100064 (MedChenExpress, HY-104070) was used at 200, 400, 600, 800, 1000 nM for 24 h; *PPARa* inhibitor GW6471 (MedChenExpress, HY-15372) was used at 0, 5, 10 µM for 48 h; *PPARa* agonist Pirinixic acid (MedChenExpress, HY-16995) was used at 0, 10, 20, 40, 60, 100 µM for 24 h.

### In vivo experiment for L-carnitine

2*10^6^ Hep3B or HCC-LM3 cells stably transfected with GSTK1(overexpressed or knockdown) cells were subcutaneously injected into the bilateral groin of 6-week-old male BALB/C-Nude mice (*n* = 6). After the injection, L-carnitine (MedChenExpress, HY-B0399) was added or not to their drinking water to obtain a final concentration of 4.0 mg/ml for 28 days until sacrificed. All animal experiments were approved by the Animal Ethics Committee of Anhui medical university.

### In vivo experiment for bexarotene

2*10^6^ Hep3B cells stably transfected with overexpressed GSTK1 were subcutaneously injected into the bilateral groin of 6-week-old male BALB/c-Nude mice (*n* = 6). 5 days after injection, Bexarotene (100 mg/kg by gavage, [MedChenExpress, HY-14171]) was used every day for in vivo experiments. Tumor xenografts were harvested and weighted at the 28 days. All animal experiments were approved by and the Animal Ethics Committee of Anhui medical university.

### In vitro tumor proliferation experiment

#### CCK8 and EdU assay

A CCK8 kit (Vazyme, A311) and a EdU kit (Beyotime, C0071) were used to access the viability of HCC cells. For CCK8 detection, cells (1*10^3^) were firstly inoculated into each well of 96-well plates, and 10 µL of CCK8 reagent was added to each well after 24,48,72 and 96 h of culture. For EdU assay, cells (1*10^5^) were seeded into each well of 24-well plates, and tested according to the manufacturer’s instruction. Images were captured with a microscopy.

#### Clone formation assay

To detect the chlorogenic ability, 500 cells were transplanted into each well of a 6-well plate, then cultured for 12–14 days and washed with PBS for every three days. Formed colonies were fixed with 4% paraformaldehyde (Servicebio, G1101) and stained with 0.1% crystal violet (Beyotime, C0121). All experiments results were from at least three separate experiments.

### In vivo tumor proliferation experiment

1*10^6^ HepG2 or HCC-LM3 cells stably transfected with GSTK1(overexpressed or knockdown) were subcutaneously injected into the bilateral groin of 6-week-old male BALB/c-Nude mice (*n* = 6). Tumor xenografts were harvested and weighted after 4 weeks. All animal experiments were approved by the Animal Ethics Committee of The Affiliated Drum Tower Hospital of Nanjing University Medical School and the Animal Ethics Committee of Anhui medical university. The approval number is ICAUC-2,502,011.

### In vitro tumor migration experiment

Migration assays were performed with a Transwell system (Millcell, Germany) coated above the membrane. Cells were suspended in pure DMEM at a concentration of 2*10^5^/ml, and then 100 µl cell suspension was loaded in the upper chamber, while the lower chamber was filled with 600ul DMEM containing 10% FBS as a chemoattractant stimulus. After incubation for 48 h, successfully translocated cells were fixed with 4% paraformaldehyde for 30 min at room temperature and stained with 0.1% crystal violet for 15 min, and then observed under light microscopy (Leica, Germany). The average cell number was calculated according to five random visions. All experiments results were from at least three separate experiments.

### Flow cytometry

Annexin V-FITC/PI Apoptosis Detection Kit (Vazyme, A211) was used to detect apoptosis, respectively. Cells (5*10^5^) were seeded into six-well plates and waited for cell attachment. After cultured with pure medium at 37℃ for 48 h, digested with non-EDTA trypsin, cells were harvested and stained with Kit for 10 min. Samples were acquired and recorded in a FACS Aria II Cell Sorter (BD Biosciences, USA), and data were analyzed with FlowJo software (TreeStar, USA).

### Reactive oxygen species (ROS) detection

Reactive Oxygen Species Assay Kit (Beyotime, S0033) was used to detect ROS. Cells treated or not treated with L-carnitine for 48 h, then washed with PBS. After digested with non-EDTA trypsin, cells were harvested and stained with DCFH-DA (Concentration at 1:1000) for 20 min at 37℃. Invert and mix every 3–5 min so that the probe is in full contact with the cells. After staining was completed, the cells were washed three times with PBS to fully remove DCFH-DA that had not entered the cells. Samples were acquired and recorded in a FACS Aria II Cell Sorter (BD Biosciences, USA), and data were analyzed with FlowJo software (TreeStar, USA).

### Transmission electron microscopy (TEM)

Related cells were collected with fixative (Servicebio, G1102) after removing the culture DMEM. After centrifugation, cells were suspended with fixative again and stored at the room temperature for 2 h, then transferred to the refrigerator at 4℃. Samples were taken photos by Transmission Electron Microscope.

### Cell ATP measurement

Cells ATP were measured by CellTiter-Lumi™ II Luminescence assay cell viability test kit (Beyotime, C0056). Remove the cell culture plate from the incubator and allow it to equilibrate at room temperature for 10 min. Then, add an equal volume of CellTiter-Lumi™ II luminescence assay reagent to the plate and incubate at room temperature with shaking for 2 min to promote cell lysis. Incubate at room temperature (approximately 25 °C) for 10 min to allow the luminescence signal to stabilize, and use a multi-functional enzyme-linked immunosorbent assay (ELISA) reader with luminescence detection capability (TECAN, Swiss) to perform the luminescence assay.

### RNA isolation and quantitative real-time PCR (RT-qPCR)

Total RNA was extracted from tissues, cancer cell lines and primary hepatocytes by using FastPure Cell/Tissue Total RNA Isolation Kit (Vazyme, RC112), according to the manufacturer’ protocol, and were reverse transcribed using the HiScript II Q RT SuperMix (+ gDNA wiper) (Vazyme, R323) for qRT-PCR. The ChamQ SYBR qPCR Master Mix) (Vazyme, Q711) was used to perform qRT-PCR on the Applied Biosystems QuantStudio 5 (ThermoFisher Scientific, USA). The primer sequences were listed below. Human *GSTK1*: Forward: TCTGGAAAAGATCGCAACGC, Reverse: GCCCAAAGGCTCCGTATCTG; Human *PPARa*: Forward: ATGGTGGACACGGAAAGCC, Reverse: CGATGGATTGCGAAATCTCTTGG; Human *RXRa*: Forward: ATGGACACCAAACATTTCCTGC, Reverse: GGGAGCTGATGACCGAGAAAG; Human *CPT1A*: Forward: TCCAGTTGGCTTATCGTGGTG, Reverse: TCCAGAGTCCGATTGATTTTTGC. Human *CPT2*: Forward: CATACAAGCTACATTTCGGGACC, Reverse: AGCCCGGAGTGTCTTCAGAA; Human *BBOX*: Forward: CTCCAGCTACCCACTTTGGAT, Reverse: CCGGTGAGTCTTACTATGCCTAC. Human *CACT*: Forward: GACACGGTCAAGGTCCGAC, Reverse: GCAGCCATTCCCCGATATAGC; Human *MFN1*: Forward: TGGCTAAGAAGGCGATTACTGC, Reverse: TCTCCGAGATAGCACCTCACC; *Human MFN2*: Forward: CTCTCGATGCAACTCTATCGTC, Reverse: TCCTGTACGTGTCTTCAAGGAA; Human *OPA1*: Forward: TGTGAGGTCTGCCAGTCTTTA, Reverse: TGTCCTTAATTGGGGTCGTTG; Human *DRP1*: Forward: ACAGTGCCAAGTTAGATGCCG.

Reverse: TCCTTGACCCTCACTAATTCCA; Human *ACTB*: Forward: CATGTACGTTGCTATCCAGGC, Reverse: CTCCTTAATGTCACGCACGAT; Mouse *Cpt1a*: Forward: CTCCGCCTGAGCCATGAAG, Reverse: CACCAGTGATGATGCCATTCT; Mouse *Cpt2*: Forward: CAGCACAGCATCGTACCCA, Reverse: TCCCAATGCCGTTCTCAAAAT; Mouse *Bbox*: Forward: ATGGGGCTCATTTGATGCAGA, Reverse: GAAGTTTCCGAGCTTTTGCAG; Mouse *Cact*: Forward: GACGAGCCGAAACCCATCAG, Reverse: AGTCGGACCTTGACCGTGT; Mouse *Fga*: Forward: AGTCTGGACTACAGATACCGAAG, Reverse: CGTCAATCAACCCTTTCATCCTG; Mouse *Gpc3*: Forward: CAGCCCGGACTCAAATGGG, Reverse: CAGCCGTGCTGTTAGTTGGTA; Mouse *Afp*: Forward: CTTCCCTCATCCTCCTGCTAC, Reverse: ACAAACTGGGTAAAGGTGATGG; Mouse *Gapdh*: Forward: AGGTCGGTGTGAACGGATTTG, Reverse: TGTAGACCATGTAGTTGAGGTCA.

### Immunoblotting analysis

Proteins from HCC cells and liver samples were extracted using ice-cold Western and IP lysis buffer (Beyotime, P0013) containing fresh protease and phosphatase inhibitors (Beyotime, P1045). And the protein concentration was measured by BCA assay (Beyotime, P0012). After denaturation with 5x loading buffer (CWBIO, CW0028) under high temperature, the protein samples were separated by SDS-PAGE and transferred to PVDF membrane, followed by immunoblotting with antibodies listed below. Rabbit antibody to Phospho-DRP1 (Ser616) (3455 [N/A] and 4494 [RRID: AB_11178659]), Mitofusin-1 (14739 [RRID: AB_2744531]), Mitofusion-2 (11925 [RRID: AB_2750893]), SQSTM1/P62 (39749 [AB_2799160]), LC3A/B (12741 [RRID: AB_2617131]), Bcl-xL (2764 [RRID: AB_2228008]), IgG Isotype Control (3900 [RRID: AB_1550038]), DYKDDDDK Tag (14793 [RRID: AB_2572291]) and mouse antibody to Bcl-2 (15071 [RRID: AB_2744528]) were obtained from Cell Signaling Technology. Rabbit antibody to GSTK1 (ab134173 [RRID: AB_1580730]), MLKL (ab184718 [RRID: AB_2755030]), DRP1 (ab184247 [RRID: AB_2895215]) and mouse antibody to CPT1A (ab128568 [RRID: AB_11141632]) were obtained from Abcam. Rabbit antibody to CPT1A (15184-1-AP [RRID: AB_2084676]), CPT2 (26555-1-AP [RRID: AB_2880551]), BBOX1 (16099-1-AP [RRID: AB_2243498]), SLC25A20 (19363-1-AP [RRID: AB_10642001]), GSTK1 (14535-1-AP [RRID: AB_2115910]), PGAM5 (28445-1-AP [RRID: AB_2881143]) and mouse antibody to PGC1-α (66369-1-Ig [RRID: AB_2828002]), PGAM5 (68116-1-Ig [RRID: AB_2923645]) and beta-actin (66009-1-Ig [RRID: AB_2687938]) were obtained from Proteintech. Secondary antibody Anti-mouse IgG (7076 [RRID: AB_330924]) and Anti-rabbit IgG (7074 [RRID: AB_2099233]) were obtained from Cell Signaling Technology. Normalization and densitometry were performed with Image Lab software (Bio-Rad, USA).

### Co-Immunoprecipitation (Co-IP)

Cells were firstly washed with PBS and then ruptured with Western and IP lysis buffer (Beyotime, P0013). After incubation on ice for 30 min, cells were centrifugated at 12,000 rpm for 15 min. Proteins were either directly analyzed by western blotting as input or used for immunoprecipitation analysis. For immunoprecipitation analysis, the proteins were incubated with corresponding antibody and mixed with Protein A + G beads (Vazyme, PB101) overnight at 4℃. The next day, after the final wash, the beads were resuspended and heated in 1X loading buffer (CWBIO, CW0028) and the supernant was further analyzed through SDS-PAGE.

### Immunohistochemistry (IHC) analysis

The immunohistochemical analysis was performed with paraffin section by the UltraSensitive SP (Mouse/Rabbit) IHC Kit (Maixin Biotech, KIT-9730). Briefly, 4 μm thick tissue sections were deparaffinized and rehydrated through xylene and graded alcohols. Antigen retrieval was performed using Citrate Solution through microwave oven at high temperature for 5 min and then low temperature for 15 min. Slides were then rinsed with PBS, marked with PAP pen. Slides were incubated with 3% hydrogen peroxide and blocking buffer both for 30 min respectively then primary antibody overnight at 4℃. Following a wash step, biotin conjugated secondary antibody and Streptavidin conjugated tertiary antibody was added sequentially for 30 min and 20 min, respectively, at room temperature and then antigens detected with 3.30-diaminobenzidine (DAB) solution (Maixin Biotech, DAB-0031).

### Multiplexable immunofluorescent staining

Multiplexable Immunofluorescent Staining was carried out by professional kit (PANOVUE, China). Briefly, paraffin-embedded tissue specimen was first dewaxed at 60℃ for 1 h. After antigen retrieval and blocking, tissue sections were covered with corresponding primary antibody overnight at 4℃, followed by incubation with poly-HRP-conjugated secondary antibody and TSA-PPD reagent. Finally, the HRP reaction was stopped through microwave-mediated antigen retrieval, and the tissue sections were processed for second and third signal detection. The nuclei were counterstained with DAPI. Images were captured using an inverted confocal fluorescent microscope.

### Liquid chromatography, and high-throughput mass spectrometry (LC-MS/MS)

Proteins from cell lysis (HepG2 overexpressing GSTK1) were immunoprecipitated with anti-GSTK1 antibody, separated by SDS-PAGE, and visualized with Coomassie brilliant blue staining eventually. Protein in-gel digestion and LC-MS/MS were carried out by APPLIED PROTEIN TECHOLOGY (ShangHai, China). The acquired MS/MS spectra were searched against the National Center for Biotechnology Information nonredundant protein sequence database.

### RNA sequencing and differentially expressed genes analysis

1. For DEN/CCl_4_ mice model RNA-seq: Total RNA from *Gstk1*^*F/F*^ and *Gstk1*^*△Hep*^ tumor tissues were extracted with RNAiso Plus Total RNA extraction reagent and checked for a RIN number to inspect RNA integrity by an Agilent Bioanalyzer 2100. Qualified total RNA was further purified by RNAClean XP Kit and RNase-Free DNase Set. After evaluating RNA purity and quantification and assessing RNA integrity, the libraries were constructed and then sequenced on an Illumina NovaSeq 6000 platform. Differential expression analysis was performed using the R package DESeq2. Q value < 0.05 and fold change > 2 or fold change < 0.5 was set as the thresholds for significantly differentially expressed genes (DEGs). KEGG pathway enrichment analysis of DEGs was performed using R software.
2. For L-carnitine treated HepG2 cells RNA-seq: Total RNA from HepG2 Ctrl and 100mM L-carnitine treated HepG2 cells were extracted with TrizoL. The raw data obtained by sequencing were filtered and the filtered clean reads were compared to the reference sequence. Differential expression analysis of two conditions/groups was performed using the DESeq2 R package (1.26.0). The resulting *P* values were adjusted using the Benjamini and Hochberg’s approach for controlling the false discovery rate. Genes with FDR < 0.05 & |log2(foldchange)| ≥1 found by DESeq2 were assigned as differentially expressed.

### Non-target metabolomics analysis

Ultra-High Performance Liquid Tandem Chromatography Quadrupole Time of Flight Mass Spectrometry (UHPLC-QTOFMS) was analyzed by BIO-Tree.CO (CHINA). Internal standard normalization method was employed in this data analysis. The resulted three-dimensional data involving the peak number, sample name, and normalized peak area were fed to SIMCA14.1 software package (V14.1, Sartorius Stedim Data Analytics AB, Umea, Sweden) for principal component analysis (PCA) and orthogonal projections to latent structures-discriminate analysis (OPLS-DA). Principal component analysis (PCA) showed the distribution of origin data. In order to obtain a higher level of group separation and get a better understanding of variables responsible for classification, supervised orthogonal projections to latent structures-discriminate analysis (OPLS-DA) were applied. Based on the orthogonal projections to latent structures-discriminate analysis (OPLS-DA), a loading plot was constructed, which showed the contribution of variables to difference between two groups. It also showed the important variables which were situated far from the origin, but the loading plot is complex because of many variables. To refine this analysis, the first principal component of variable importance in the projection (VIP) was obtained. The VIP values exceeding 1 were first selected as changed metabolites. In step 2, the remaining variables were then assessed by Student’s t-test (*P*-value > 0.05), variables were discarded between two comparison groups. In addition, commercial databases including KEGG (http://www.genome.jp/kegg/) and MetaboAnalyst (http://www.metaboanalyst.ca/) was utilized to search for the pathways of metabolites.

### Measurement of cancer cell L-carnitine and acylcarnitine

Measurement of L-carnitine and Acylcarnitine through an Elisa kit (COIBO BIO, CB11833 [Human], CB12887 [Mus], CB13180 [Mus]). Add samples to enzyme well which has been pre-coated with antibodies, then add recognition antigen labeled by horse radish peroxidase (HRP); after been incubated 30 min at 37℃, both compete with solid phase antigen and formed immune complex; after been washing by PBST, the combined HRP catalyses TMB (Tetramethyl benzidine) into blue, and turns into yellow by the action of acid; it has absorption peak under 450 nm wavelength, and its absorbance is negatively correlated with antigen density of sample.

### Quantification and statistical analysis

Data are presented as mean, means ± SD as indicated in the figure legends. Differences in means were analyzed by Student’s t test or one-way ANOVA and Tukey’s honestly significant difference (HSD) post hoc test when the normality of data distribution and equal variance between groups were met, otherwise non-parametric tests (Wilcoxon) were applied. χ2 test and Student’s t-test analysis of variance were used to evaluate statistical differences in demographic and clinical characteristics. Spearman′s rank correlation coefficient analysis was applied to analyze the relationships between associated factors. *p* values < 0.05 were considered statistically significant (n.s. no significant, *, *p* < 0.05, **, *p* < 0.01, ***, *p* < 0.001, ****, *p* < 0.0001).

## Supplementary Information

Supplementary Material 1.

Supplementary Material 2.

## Data Availability

The data analyzed in this study were obtained from Gene Expression Omnibus at GSE14520 and HCCDB8. All other raw data are available upon request from the corresponding author.

## Abbreviations

BBOX: gamma-butyrobetaine hydroxylase
CS: carnitine system
CPT1: carnitine palmitoyl transferase 1
CPT2: carnitine palmitoyl transferase 2
CACT: carnitine/acyl-carnitine translocase
CCl_4_: carbon tetrachloride
DRP1: dynamin related protein 1
DsbA-L: disulfide-bond A oxidoreductase-like protein
EdU: 5-Ethynyl-2’-deoxyuridine
GSTK1: glutathione s-transferase kappa 1
HBV: hepatitis B virus
HCV: hepatitis C virus
HCC: hepatocellular carcinoma
HFFCD: high fat, high fructose, high cholesterol diet
IF: immunofluorescence
IP: immunoprecipitation
LC-MS/MS: liquid chromatography and high-throughput mass spectrometry
MASH: metabolic dysfunction-associated steatohepatitis
MFF: mitochondrial fission factor
MFN1: mitofusion 1
MFN2: mitofusion 2
MQC: mitochondrial quality control
NASH: non-alcoholic steatohepatitis
OS: overall survival
TEM: transmission electron microscope
PGAM5: phosphoglycerate mutase 5
PGC1-a: peroxisome proliferator-activated receptor gamma coactivator-1α
PPARα: peroxisome proliferator activated receptor alpha
PCNA: proliferating cell nuclear antigen
RXRα: retinoid x receptor alpha
RXRs: retinoid X receptors
