# Supplementary material for: GSTK1 suppresses HCC aggravation via L-carnitine metabolism by PGAM5/DRP1 complex-mediated mitochondrial quality control
Source: J Exp Clin Cancer Res. 2025 Nov 24;45:1. doi: 10.1186/s13046-025-03580-8 (PMC12763885; doi:10.1186/s13046-025-03580-8)
Supplement: Supplementary file 1 — Supplementary Material 1. [file 13046_2025_3580_MOESM1_ESM.docx]

**Title:** GSTK1 suppresses HCC aggravation via L-carnitine metabolism by PGAM5/DRP1 complex-mediated mitochondrial quality control

Authors: Yuze Shi^1,2#^, Jinyao Zhang^2#^, Bojiao Song^2#^, Haitian Zhang^2#^, Jianbo He^1,2^, Ke Ding^2^, Fei Wang^1^, Weiwei Yu^3^, Guangyan Zhangyuan^4^, Kangpeng Jin^5*^, Wenjie Zhang^2*^, Beicheng Sun^1,2*^

Supplementary Figures


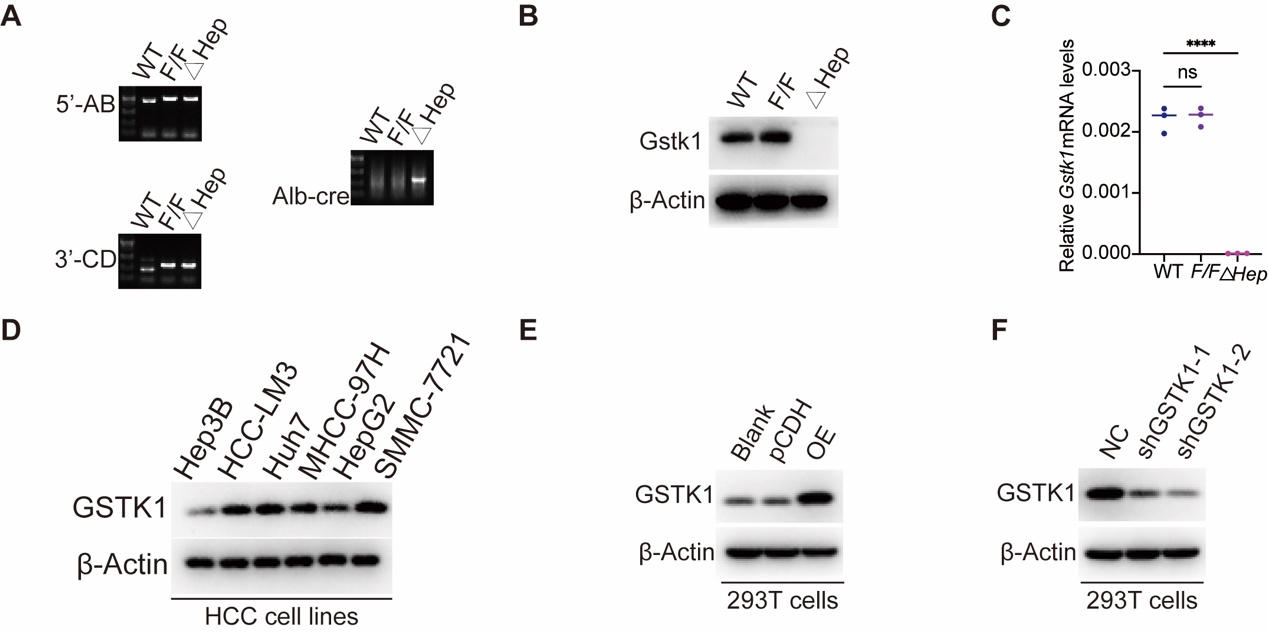


**Figure S1**. Validation of *Gstk1* liver-specific knockout mice and identification of overexpress GSTK1 plasmid or shRNA of GSTK1. (**A**) Genotyping PCR showing 5’ (left top) and 3’ (left bottom) loxP site insertion and Genotyping PCR for albumin-Cre transgenic mice (right). (**B-C**) Immunoblotting and RT-qPCR analysis of Gstk1 (*Gstk1*) in *WT*, *Gstk1^F/F^*, *Gstk1^△Hep^* mice. (**D**) Immunoblotting analysis of GSTK1 in various HCC cell lines. (**E**) Immunoblotting analysis of GSTK1 in 293T cells transfected with overexpress GSTK1 plasmid and control pCDH plasmid. (**F**) Immunoblotting analysis of GSTK1 in 293T cells transfected with shRNA of GSTK1 and control NC. Data are presented as mean ± SD. **** *p*<0.0001

**
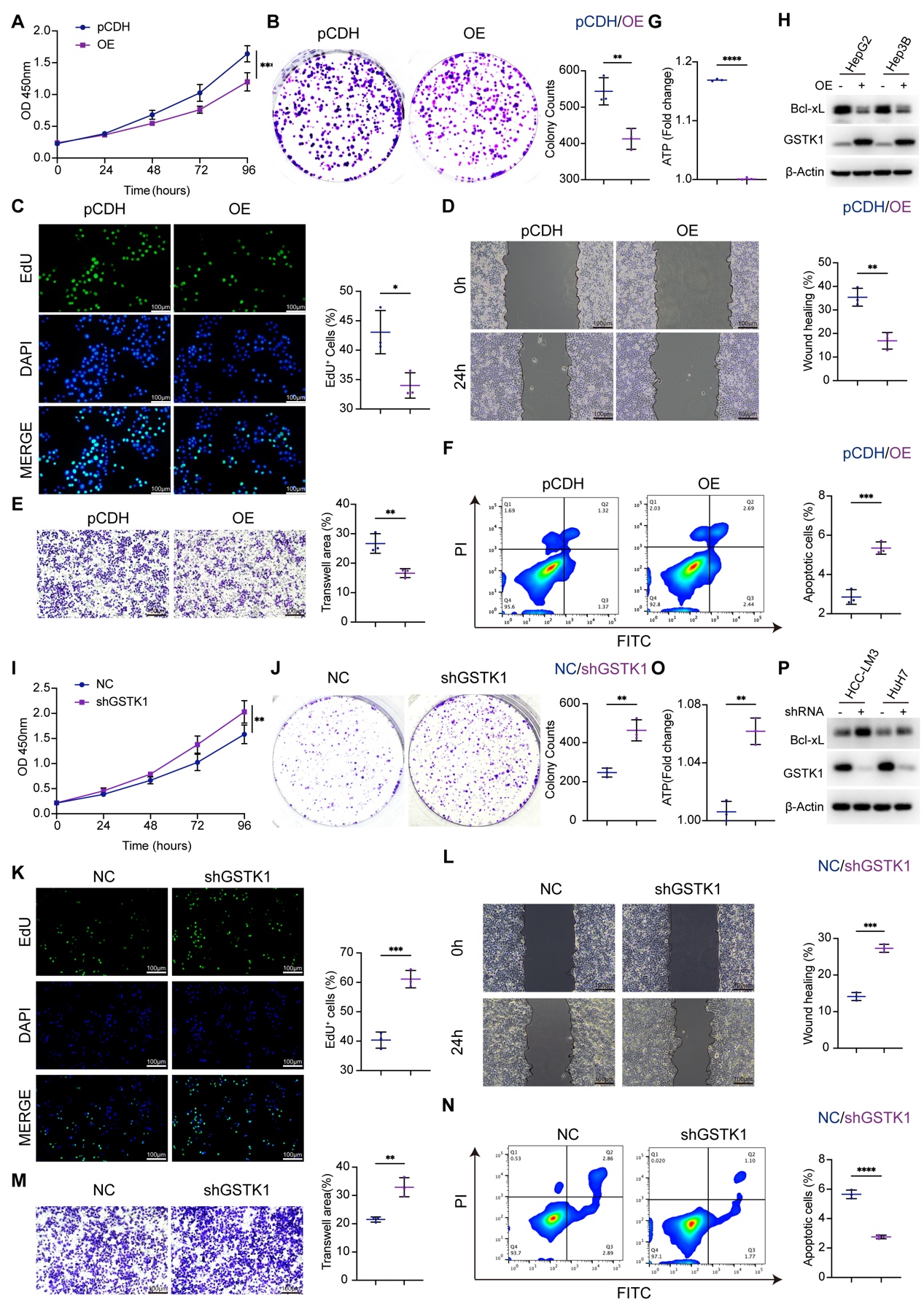
**

**Figure S2**. GSTK1 inhibited proliferation and migration in HCC cell lines. (**A-C**) Cell proliferation ability detection of Hep3B cells after overexpress GSTK1. CCK8 (**A**), Colony formation (**B**), EdU (**C**) (Bar = 100 μm). (**D-E**) Cell migration ability detection of Hep3B cells after overexpress GSTK1. Wound healing (**D**), Transwell (**E**) (Bar = 100 μm). (**F**) The apoptosis ratio of Hep3B cells after overexpress GSTK1 detected by flow cytometry using the Annexin V-FITC/PI staining kit. (**G**) The ATP measurement of Hep3B cells after overexpress GSTK1. (**H**) Immunoblotting analysis of Bcl-xL, Bcl-2 and β-Actin in HepG2 and Hep3B cells after overexpress GSTK1. (**I-K**) Cell proliferation ability detection of Huh7 cells after knockdown GSTK1. CCK8 (**I**), Colony formation (**J**), EdU (**K**) (Bar = 100 μm). (**L-M**) Cell migration ability detection of Huh7 cells after knockdown GSTK1.Wound healing(**J**), Transwell(**K**) (Bar = 100 μm). (**N**) The apoptosis ratio of Huh7 cells after knockdown GSTK1 detected by flow cytometry using the Annexin V-FITC/PI staining kit. (**O**) The ATP measurement of Huh7 cells after knockdown GSTK1. (**P**) Immunoblotting analysis of Bcl-xL, Bcl-2 and β-Actin in HCC-LM3 and Huh7 cells after knockdown GSTK1. Data are presented as mean ± SD. * *p*< 0.05, ** *p* < 0.01, *** *p* < 0.001 and **** *p*<0.0001


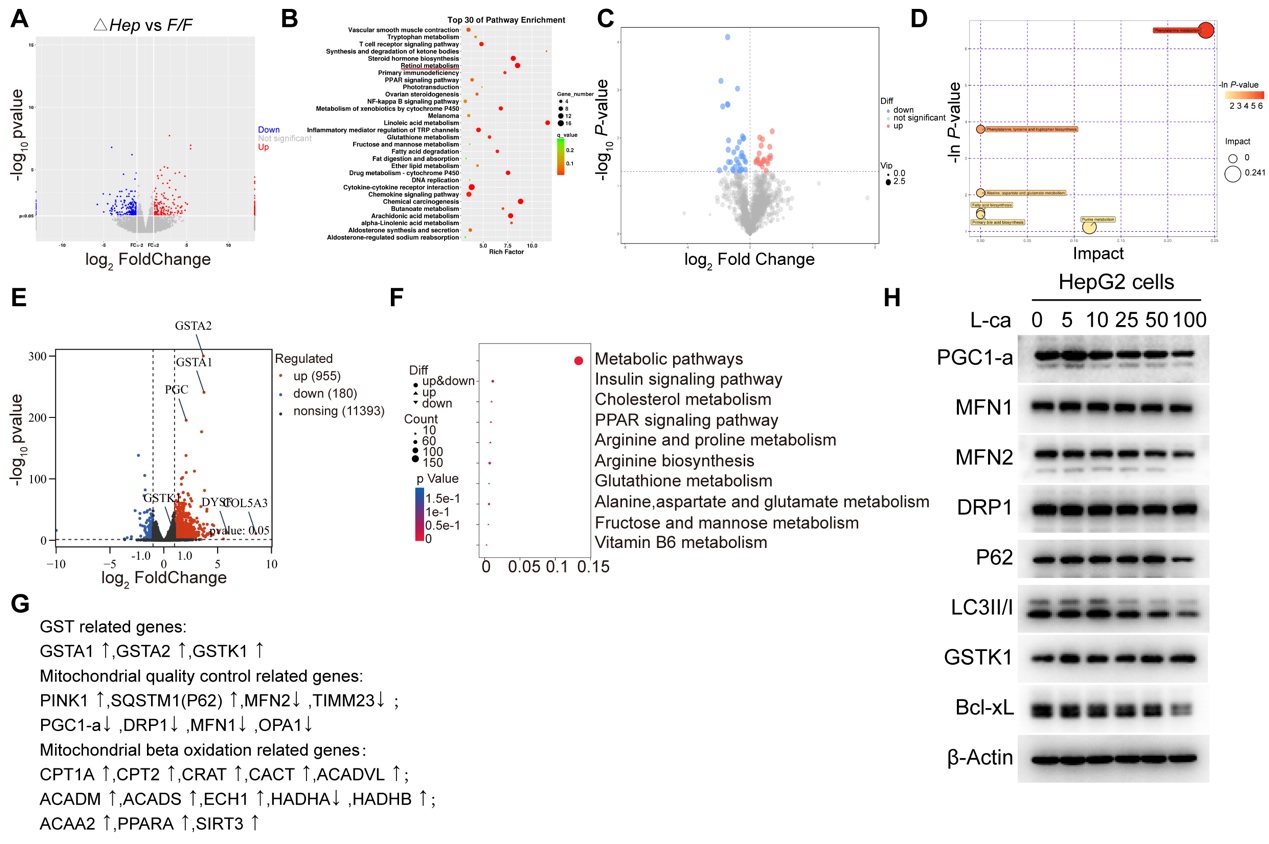


**Figure S3**. GSTK1 is responsible for L-carnitine metabolism. (**A-B**) RNA-sequence results of DEN/CCl_4_ HCC model related to **Figure 1**. (**C-D**) Nor-target metabolomics results of DEN/CCl_4_ HCC model related to **Figure 1**. (**E-F**) RNA-sequence results of Ctrl and L-carnitine treated (100 mM) HepG2 cell for 48 h. (**G**) Mitochondrial fatty acid β-oxidation, mitochondrial quality control and GSH related genes analysis of RNA-seq with Ctrl and L-carnitine treated HepG2 cells. (**H**) Immunoblotting analysis of MFN1, MFN2, DRP1, PGC1-α, P62, LC3II/I, GSTK1, Bcl-xL and β-Actin in HepG2 cells treated with gradient concentration of L-carnitine for 48 h.


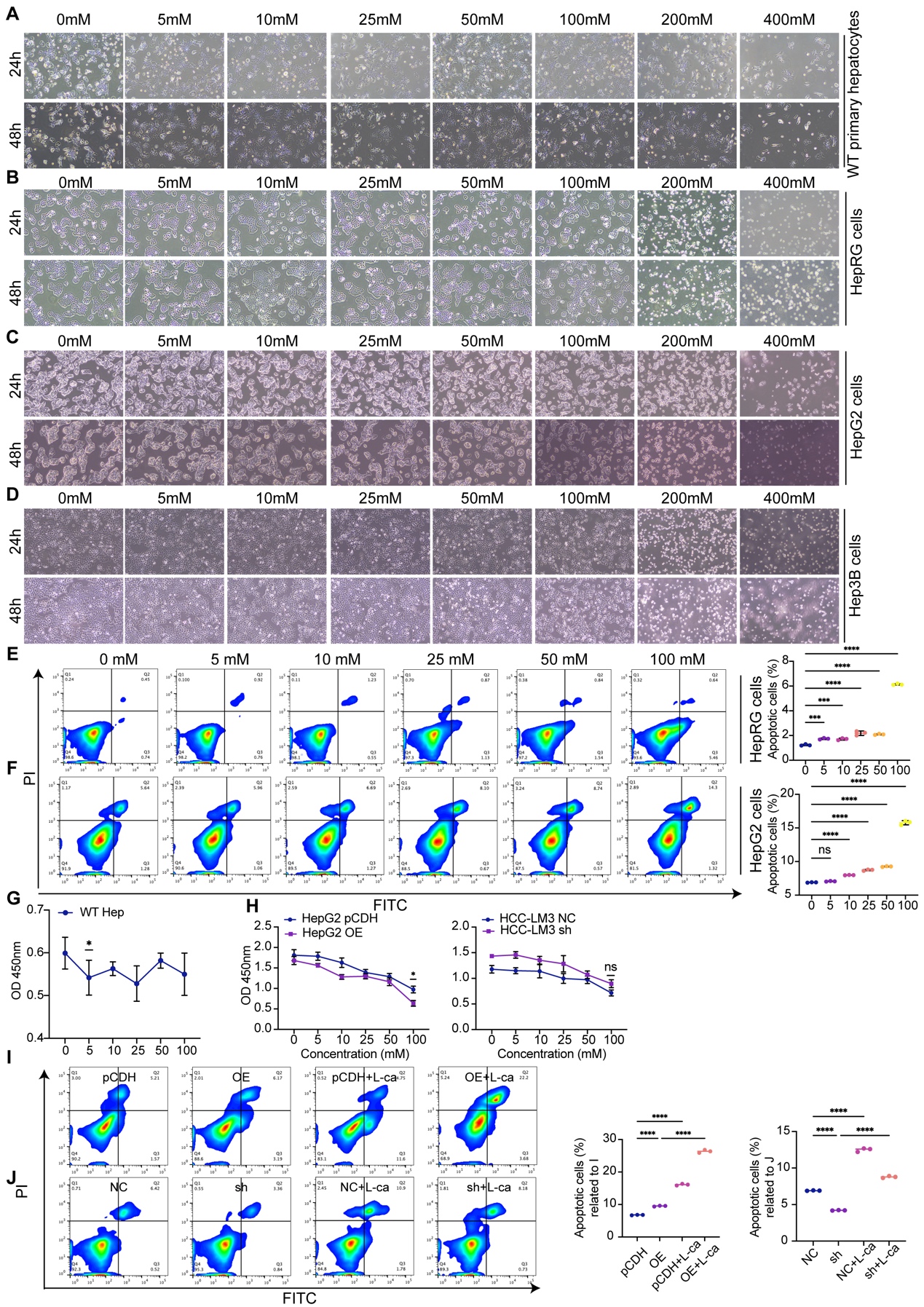


**Figure S4**. L-carnitine could against HCC cells. (**A-D**) Images of WT primary hepatocytes (**A**), HepRG (**B**), HepG2 (**C**) and Hep3B (**D**) cells treated by gradient concentration of L-carnitine for 24 h or 48 h. (**E-F**) Apoptotic ratio of cells treated by gradient concentration of L-carnitine for 48 h in HepRG (**E**) and HepG2 (**F**) cells. Statistical analysis on the Right. (**G**) Cell proliferation ability detection of WT primary hepatocytes treated by gradient concentration of L-carnitine for 48 h, detected by CCK8. (**H**) Cell proliferation ability detection of HepG2 cells after overexpress GSTK1 (**F**), HCC-LM3 cells knockdown GSTK1 (**G**) treated by gradient concentration of L-carnitine for 48 h, detected by CCK8. (**I-J**) Apoptotic ratio of HepG2 cells overexpressing GSTK1 or HCC-LM3 cells with GSTK1 knockdown following treatment with L-carnitine (100 mM) for 48 h. Data are presented as mean ± SD. n.s, no significance, * *p*< 0.05, ** *p* < 0.01, *** *p* < 0.001 and **** *p*<0.0001


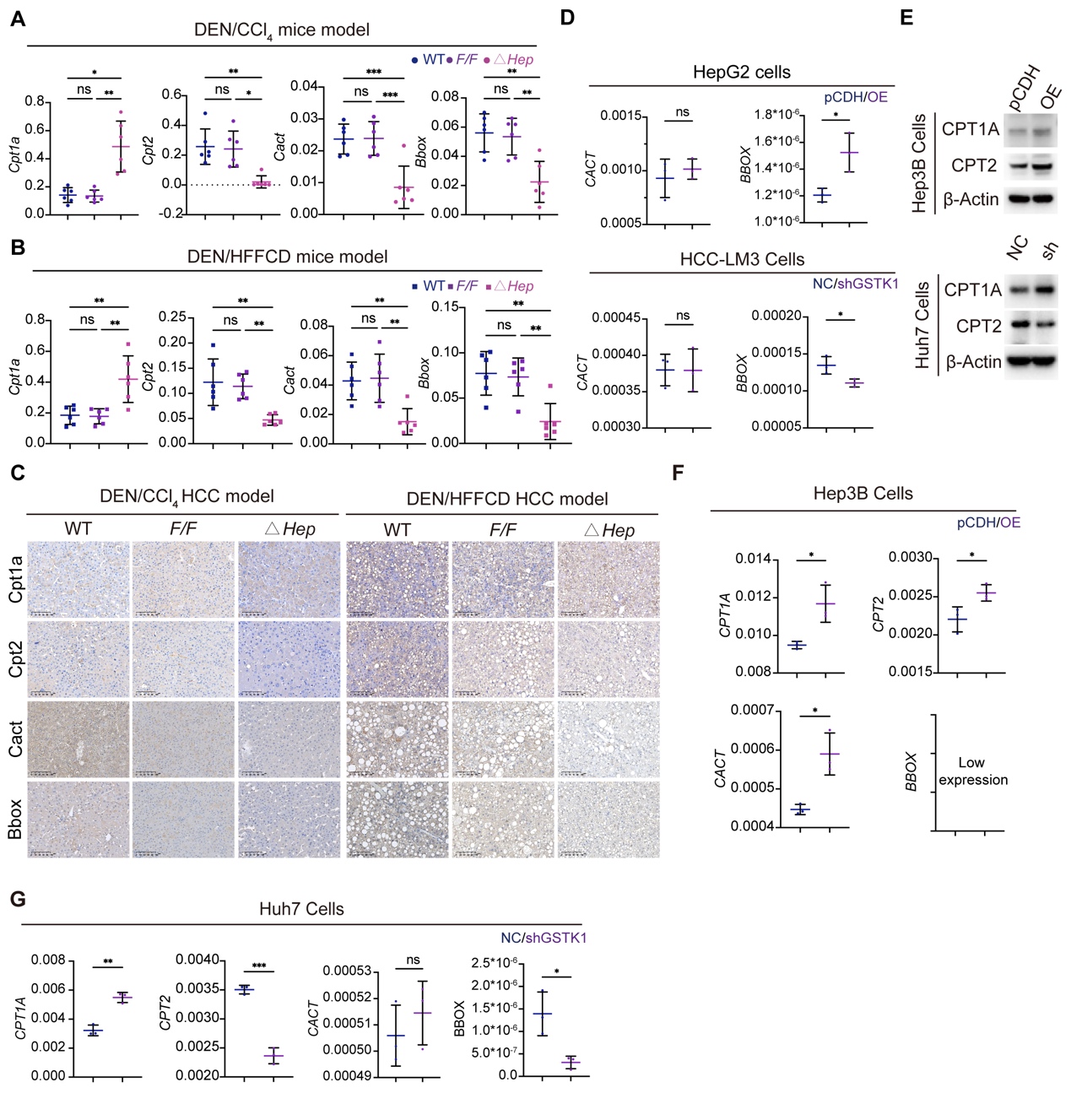


**Figure S5**. GSTK1 regulates key transporters of L-carnitine metabolism. (**A-B**) RT-qPCR analysis of *Cpt1a*, *Cpt2*, *Cact* and *Bbox* in DEN/CCl_4_ and DEN/HFFCD HCC model related to **Figure 1**. (**C**) Immunohistochemistry staining of Cpt1a, Cpt2, Cact and Bbox in DEN/CCl_4_ and DEN/HFFCD HCC model related to **Figure 1**. (**D**) RT-qPCR analysis of *CACT* and *BBOX* in HCC cell lines related to **Figure 2**. (**E**) Immunoblotting analysis of CPT1A, CPT2 and β-Actin in HCC cell lines related to **Figure S2**. (**F-G**) RT-qPCR analysis of *CPT1A*, *CPT2*, *CACT* and *BBOX* in HCC cell lines related to **Figure S2**. Data are presented as mean ± SD. n.s, no significance, * *p*< 0.05, ** *p* < 0.01.


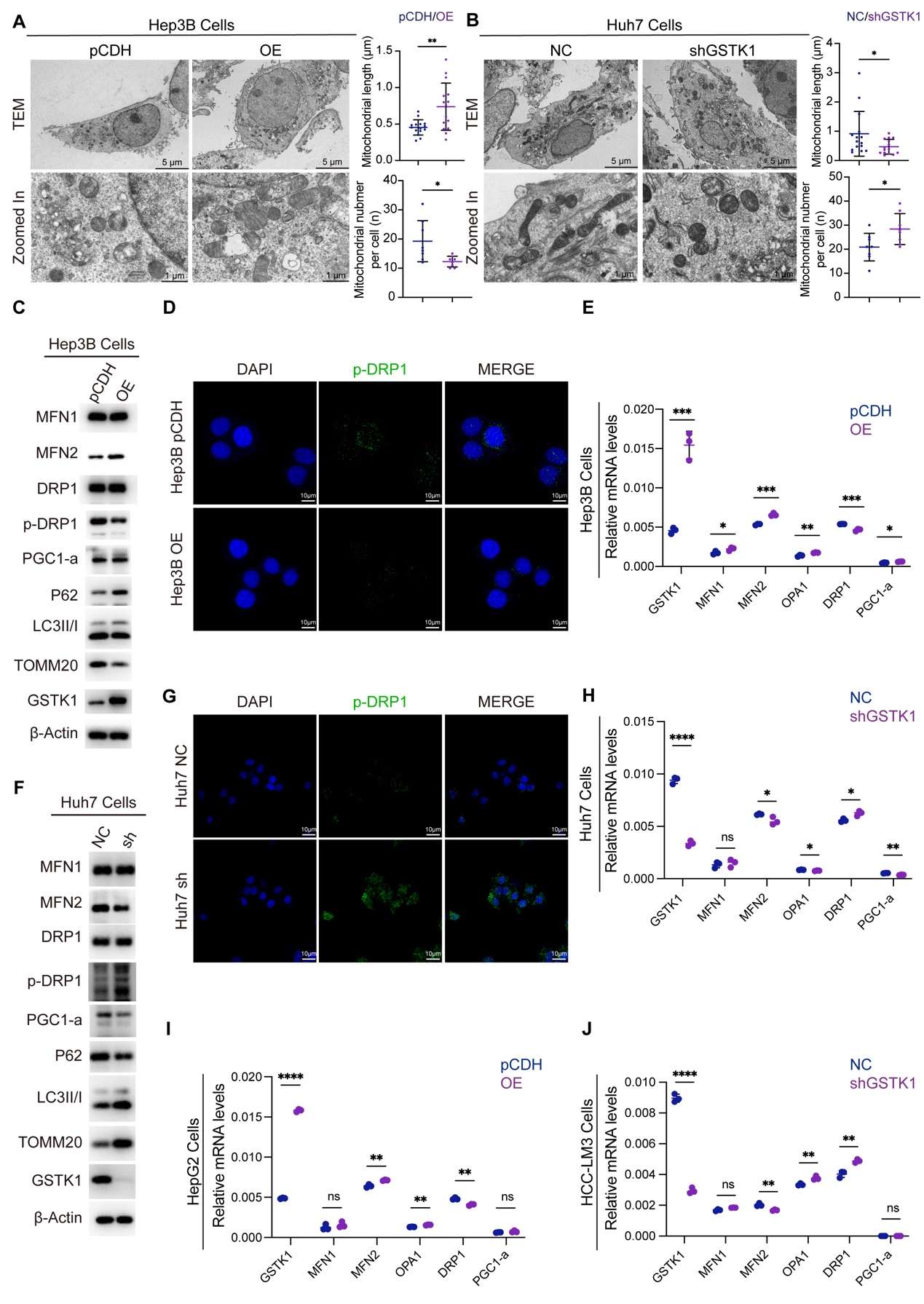


**Figure S6**. GSTK1 promotes mitochondrial biosynthesis and mitochondrial fusion, while inhibiting mitochondrial fission and mitophagy. (**A-B**) Representative TEM images of Hep3B cells overexpress GSTK1 (**A**) or Huh7 cells knockdown GSTK1 (**B**) (Bar = 5 μm or 1 μm). (**C&F**) Immunoblotting analysis of MFN1, MFN2, DRP1, p-DRP1, PGC1-α, P62, LC3II/I, TOMM20, GSTK1 and β-Actin in Hep3B cells overexpress GSTK1 (**C**) or Huh7 cells knockdown GSTK1 (**F**). (**D and G**) Immunofluorescent staining of pDRP1(S616) in Hep3B cells overexpress GSTK1 (**D**) or Huh7 cells knockdown GSTK1 (**G**) (Bar = 10 μm). (**E, H-J**) RT-qPCR analysis of GSTK1, MFN1, MFN2, OPA1, DRP1 and PGC1-a mRNA levels in Hep3B (**E**) and HepG2 (**I**) cells overexpress GSTK1 or Huh7 (**H**) and HCC-LM3 (**J**) cells knockdown GSTK1. Data are presented as mean ± SD. n.s, no significance, * *p*< 0.05, ** *p* < 0.01, *** *p* < 0.001 and **** *p*<0.0001


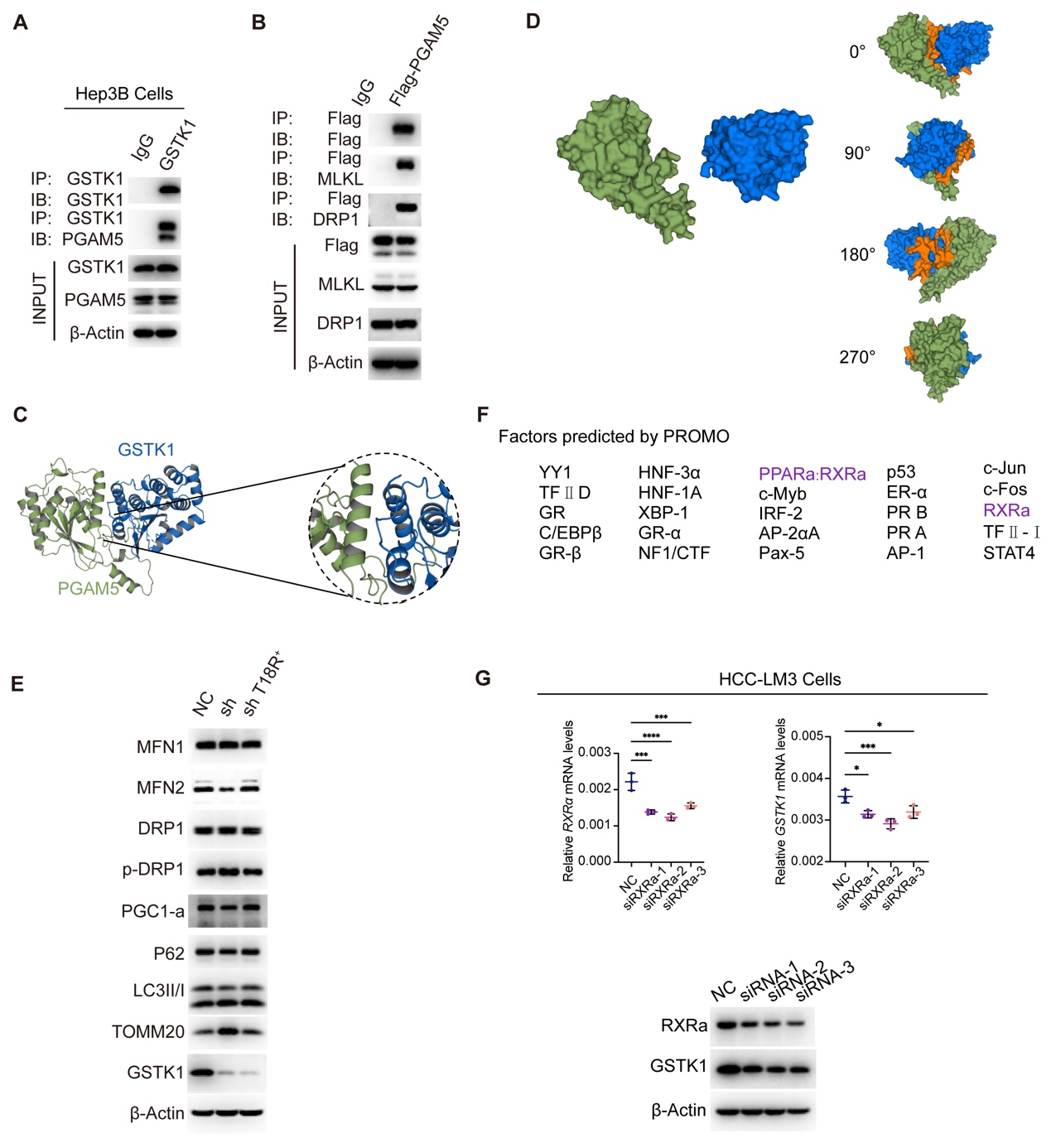


**Figure S7**. GSTK1 competes with DRP1 for binding to PGAM5, and GSTK1 is regulated by PPARα/RXRα. (**A**) Immunoblotting analysis of GSTK1 and PGAM5 interaction in Hep3B cells overexpress GSTK1. (**B**) Immunoblotting analysis of DRP1, MLKL, and Flag-PGAM5 interaction in HepG2 cells stably transfected Flag-PGAM5. (**C-D**) The prediction of specific binding sites for GSTK1 and PGAM5 interaction, detected by PLIP software. (**E**) Immunoblotting analysis of MFN1, MFN2, DRP1, p-DRP1, PGC1-α, P62, LC3II/I, TOMM20, GSTK1 and β-Actin in HCC-LM3 cells knockdown GSTK1 and with TYR-18 point mutation. (**F**) Prediction of GSTK1 transcription factors by PROMO. (**G**) Immunoblotting and RT-qPCR analysis of *RXRα* and *GSTK1* in HCC-LM3 cells transient transfection of siRXRα. Data are presented as mean ± SD. n.s, no significance, * *p*< 0.05, *** *p* < 0.001 and **** *p*<0.0001


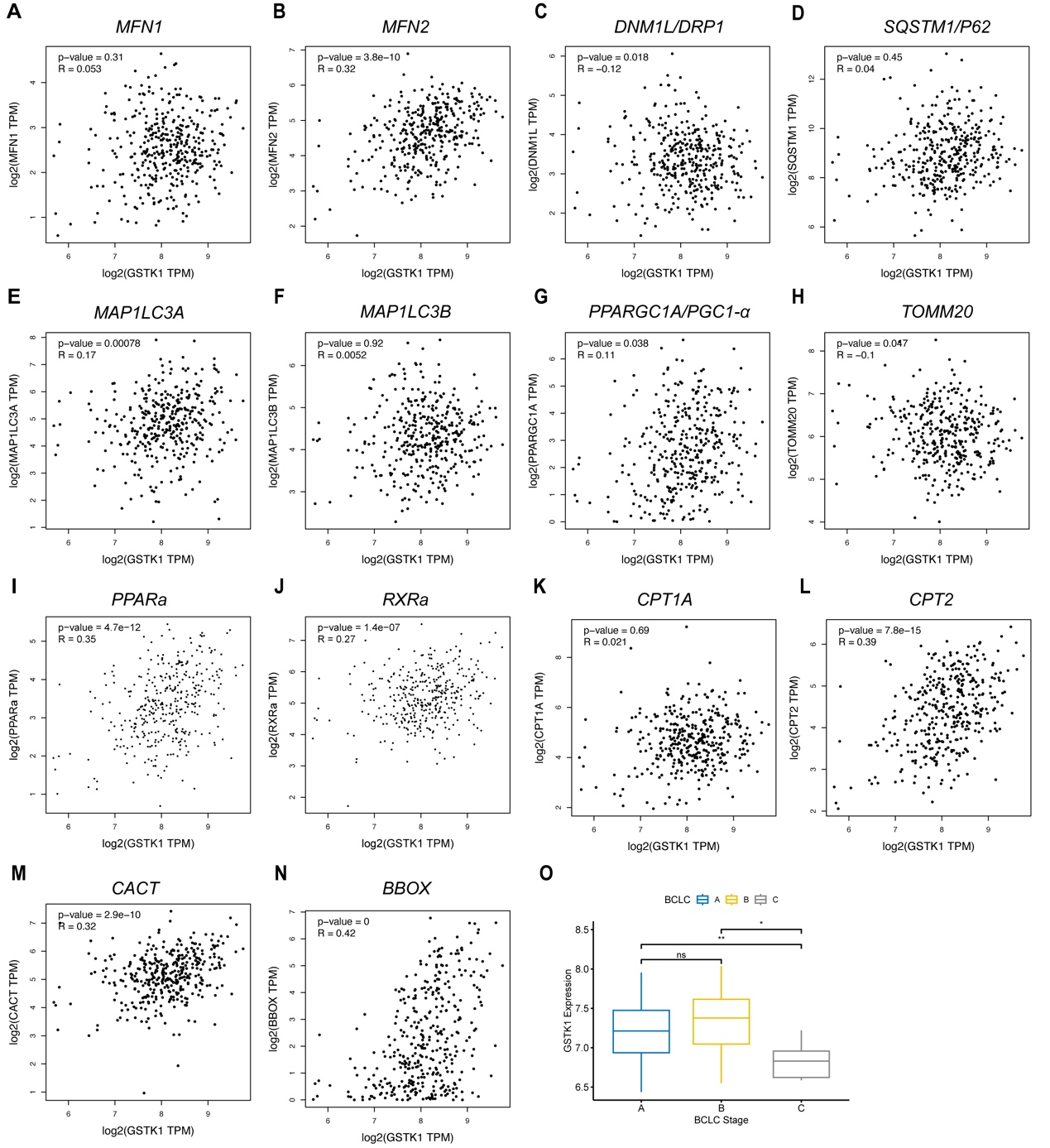


**Figure S8**. *GSTK1* and MQC related or L-carnitine metabolism Related genes or *PPARa/RXRa* correlation in HCC patients analyzed by database GEPIA2. (**A-N**) Correlation between *GSTK1* and *MFN1* (**A**), *MFN2* (**B**), *DRP1* (**C**), *P62* (**D**), *MAP1LC3A* (**E**), *MAP1LC3B* (**F**), *PGC1-α* (**G**), *TOMM20* (**H**), *PPARα* (**I**), *RXRα* (**J**), *CPT1A* (**K**), *CPT2* (**L**), *CACT* (**M**), *BBOX* (**N**) in HCC patients analyzed by database GEPIA2. (**O**) The correlation between GSTK1 expression and the BCLC staging of HCC patients (HCCDB8 cohort). n.s, no significance, * *p*< 0.05, ** *p* < 0.01

**Table. S1**. Correlation analysis between GSTK1 expression and clinical information of HCC patients(n=214)

| **Characteristic** | **Number** | **GSTK1 expression** | | ***P* value** |
| --- | --- | --- | --- | --- |
|  |  | **High** | **Low** |  |
| Metastasis risk |  |  |  |  |
| High | 103(48.13%) | 25(23.58%) | 78(72.22%) | *＜0.001* |
| Low | 111(51.87%) | 81(76.42%) | 30(27.78%) |  |
| Gender |  |  |  | 0.579 |
| Femal | 28(13.08%) | 12(11.32%) | 16(14.81%) |  |
| Male | 186(86.92%) | 94(88.68%) | 92(85.19%) |  |
| Hepatitis B virus infection |  |  |  |  |
| AVR-CC | 55(25.7%) | 19(17.92%) | 36(33.33%) | *0.035* |
| CC | 153(71.5%) | 84(79.25%) | 69(63.89%) |  |
| NA | 6(2.8%) | 3(2.83%) | 3(2.78%) |  |
| ALT |  |  |  |  |
| HIgh(>50U/L) | 87(40.65%) | 42(39.62%) | 45(41.67%) | 0.869 |
| Low(<=50U/L) | 127(59.35%) | 64(60.38%) | 63(58.33%) |  |
| Tumor size |  |  |  |  |
| >5 cm | 76(35.51%) | 34(32.08%) | 42(38.89%) | 0.369 |
| <=5 cm | 138(64.49%) | 72(67.92%) | 66(61.11%) |  |
| Multiple nodules |  |  |  |  |
| No | 169(78.97%) | 93(87.74%) | 76(70.37%) | *0.003* |
| Yes | 45(21.03%) | 13(12.26%) | 32(29.63%) |  |
| Cirrhosis |  |  |  |  |
| No | 18(8.41%) | 11(10.38%) | 7(6.48%) | 0.435 |
| Yes | 196(91.59%) | 95(89.62%) | 101(93.52%) |  |
| TNM staging |  |  |  |  |
| I | 89(41.59%) | 52(49.06%) | 37(34.26%) | *0.004* |
| II | 77(35.98%) | 40(37.74%) | 37(34.26%) |  |
| III | 48(22.43%) | 14(13.21%) | 34(31.48%) |  |
| BCLC staging |  |  |  |  |
| 0 | 20(9.35%) | 10(9.43%) | 10(9.26%) | *0.003* |
| A | 143(66.82%) | 82(77.36%) | 61(56.48%) |  |
| B | 22(10.28%) | 5(4.72%) | 17(15.74%) |  |
| C | 29(13.55%) | 9(8.49%) | 20(18.52%) |  |
| CLIP staging |  |  |  |  |
| 0 | 95(44.39%) | 69(65.09%) | 26(24.07%) | *＜0.001* |
| 1 | 71(33.18%) | 24(22.64%) | 47(43.52%) |  |
| 2 | 35(16.36%) | 8(7.55%) | 27(25%) |  |
| 3 | 9(4.21%) | 3(2.83%) | 6(5.56%) |  |
| 4 | 3(1.4%) | 2(1.89%) | 1(0.93%) |  |
| 5 | 1(0.47%) | 0(0%) | 1(0.93%) |  |
| AFP |  |  |  |  |
| High(>300ng/ml) | 96(44.86%) | 26(24.53%) | 70(64.81%) | *＜0.001* |
| Low(<=300ng/ml) | 118(55.14%) | 80(75.47%) | 38(35.19%) |  |

NA, not available; AVR-CC, active viral replication chronic carrier; CC, chronic carrier.
