# Supplementary material for: GSTK1 suppresses HCC aggravation via L-carnitine metabolism by PGAM5/DRP1 complex-mediated mitochondrial quality control
Source: J Exp Clin Cancer Res. 2025 Nov 24;45:1. doi: 10.1186/s13046-025-03580-8 (PMC12763885; doi:10.1186/s13046-025-03580-8)

Figure 3

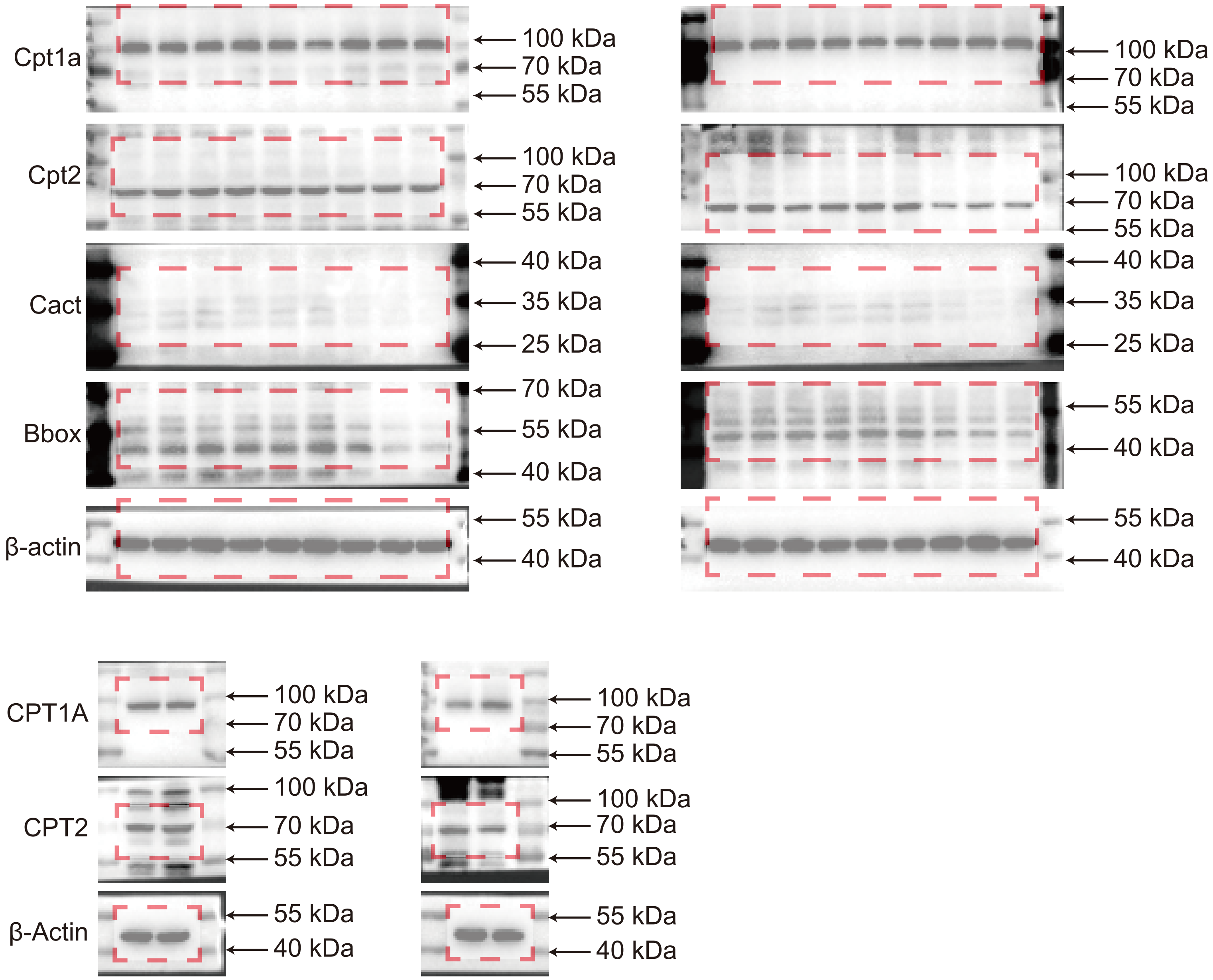
Cpt1a
100 kDa
70 kDa
55 kDa
Cpt2
Cact
40 kDa
35 kDa
25 kDa
Bbox
β-actin
CPT1A
CPT2
β-Actin

Figure 4C

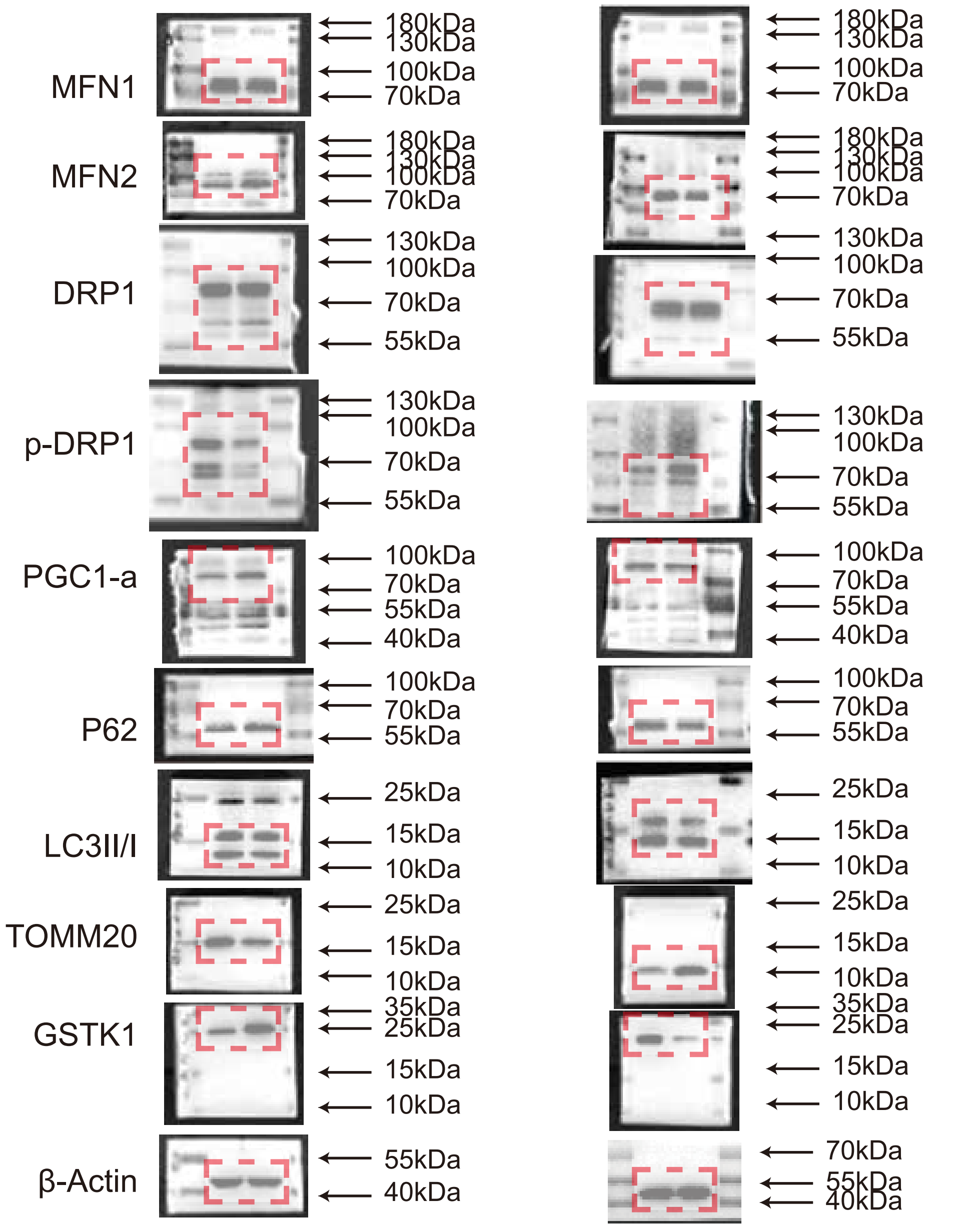

Figure 5F

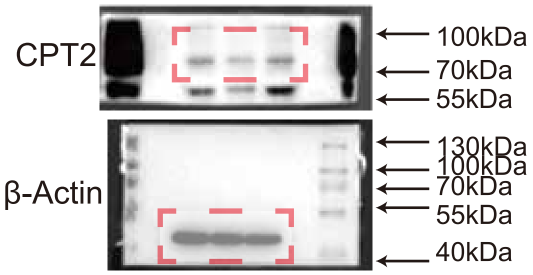

Figure 4F

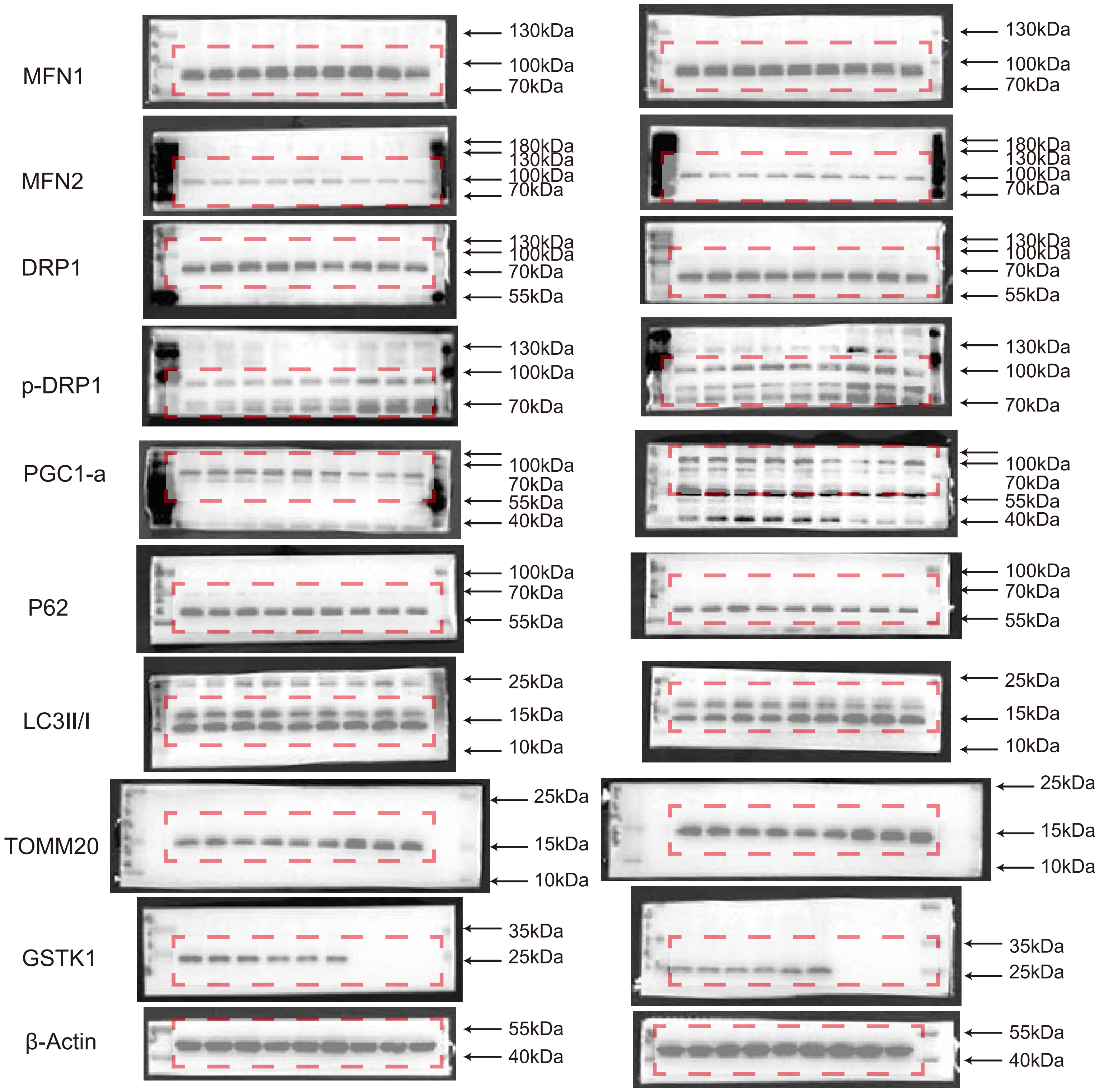
MFN1
130kDa
100kDa
70kDa
MFN2
180kDa
130kDa
100kDa
70kDa
DRP1
130kDa
100kDa
70kDa
55kDa
p-DRP1
130kDa
100kDa
70kDa
PGC1-a
100kDa
70kDa
55kDa
40kDa
P62
100kDa
70kDa
55kDa
LC3II/I
25kDa
15kDa
10kDa
TOMM20
25kDa
15kDa
10kDa
GSTK1
35kDa
25kDa
β-Actin
55kDa
40kDa

Figure 6C

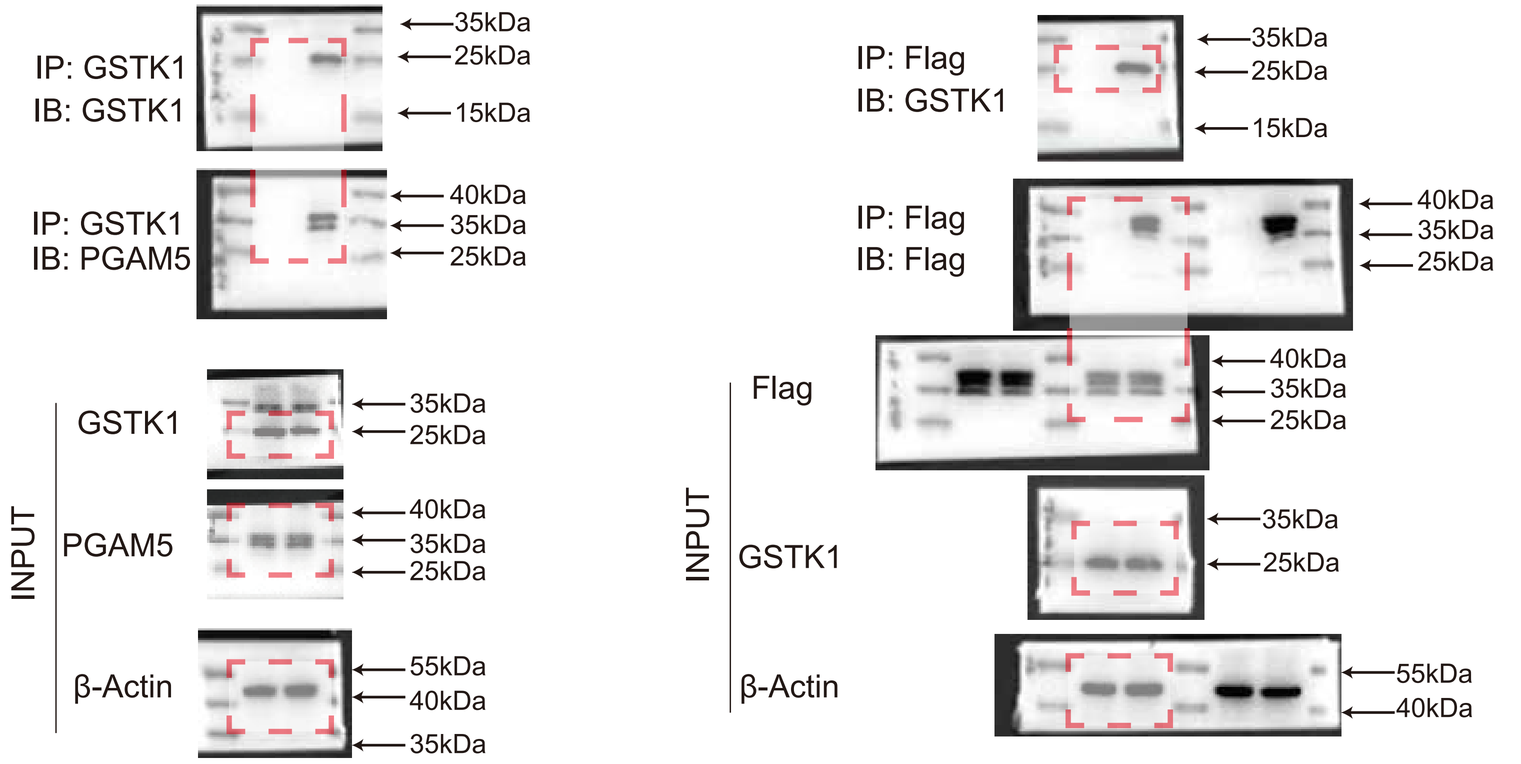

Figure 6E

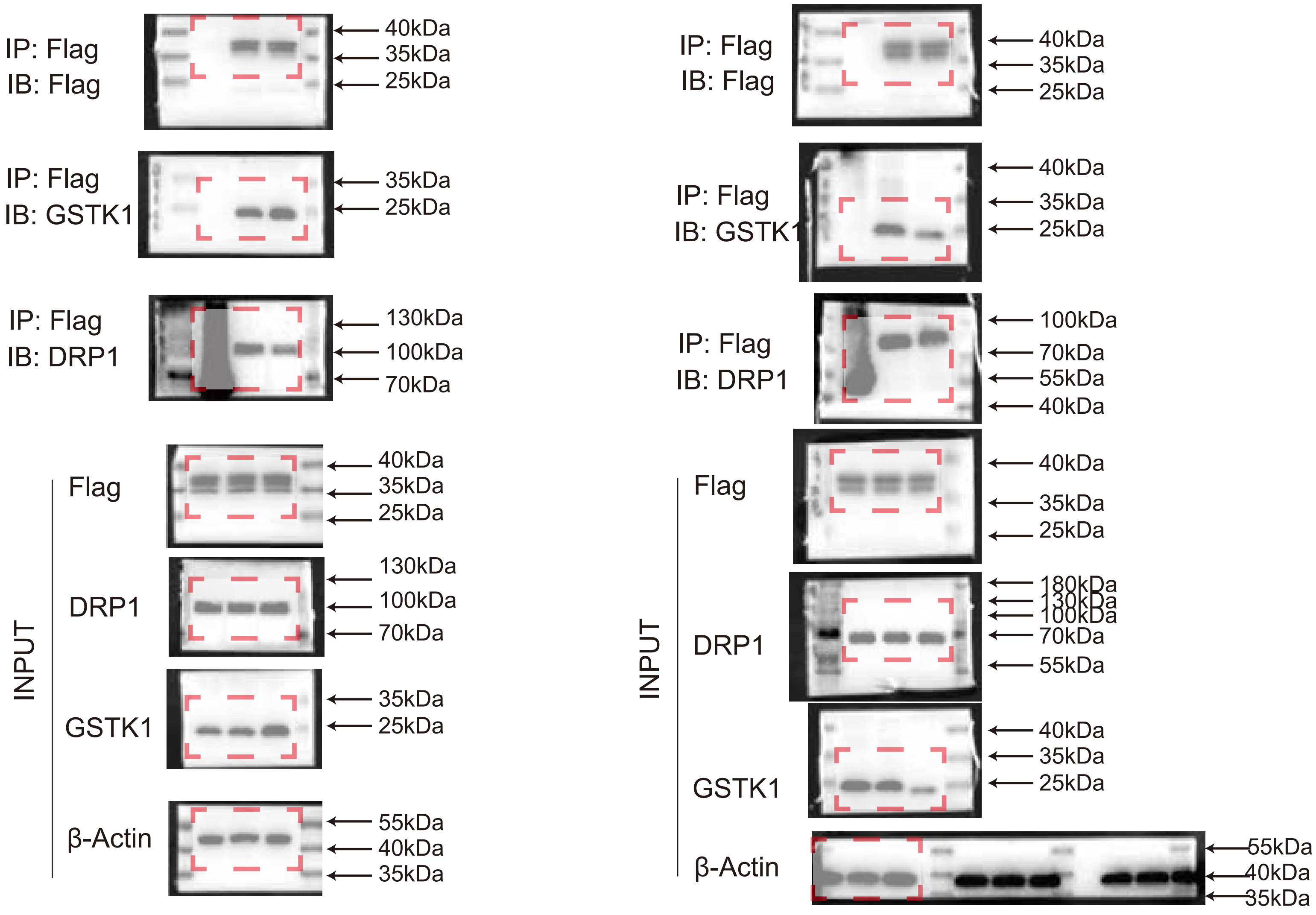

Figure 6G

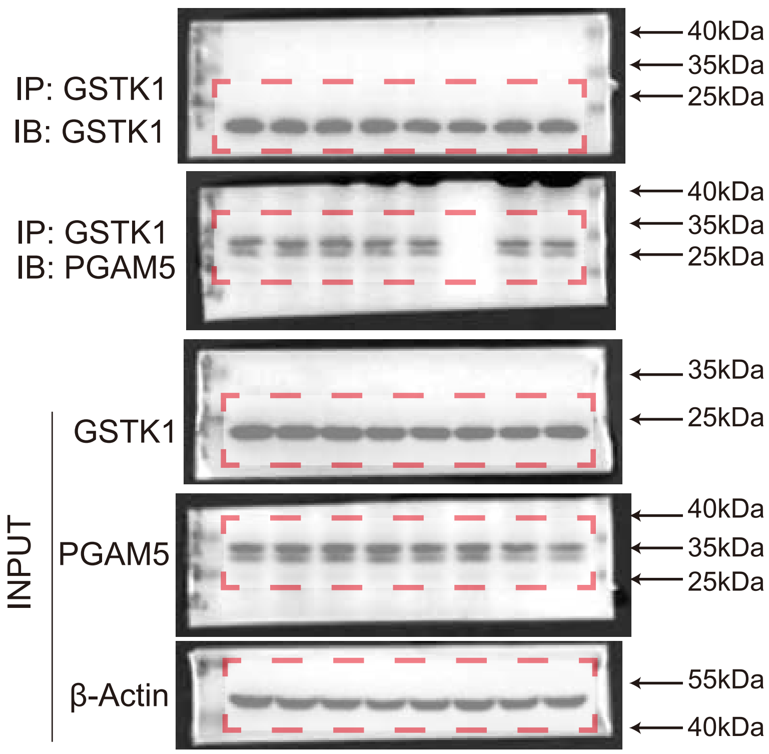

Figure 6H

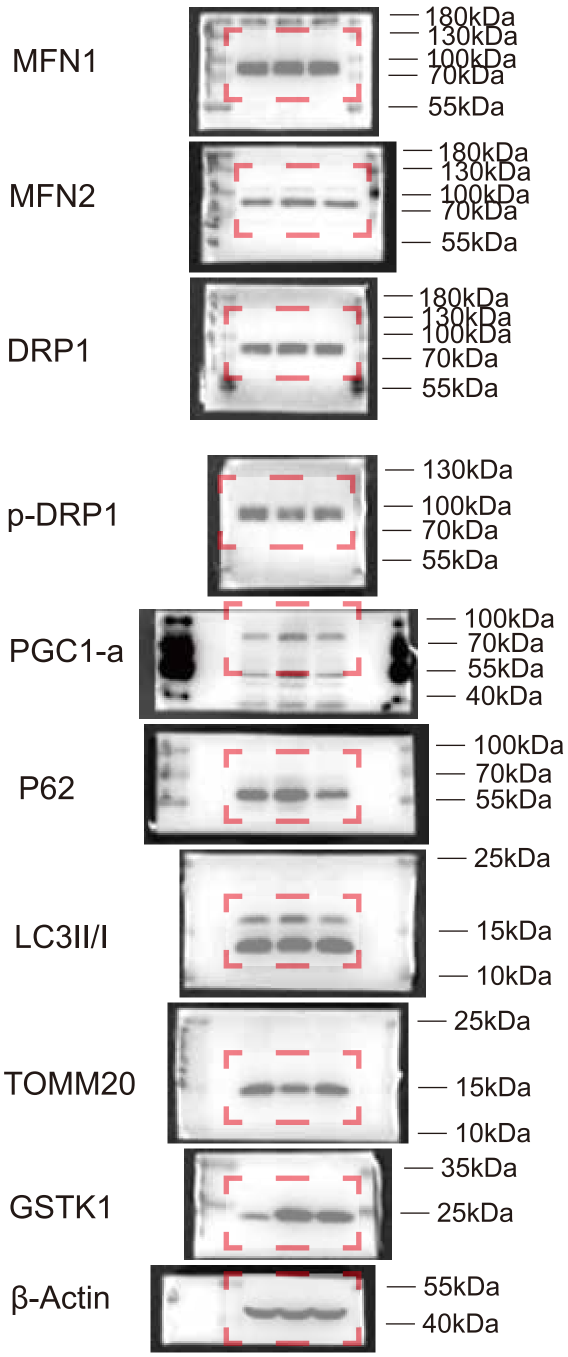

Figure 7M

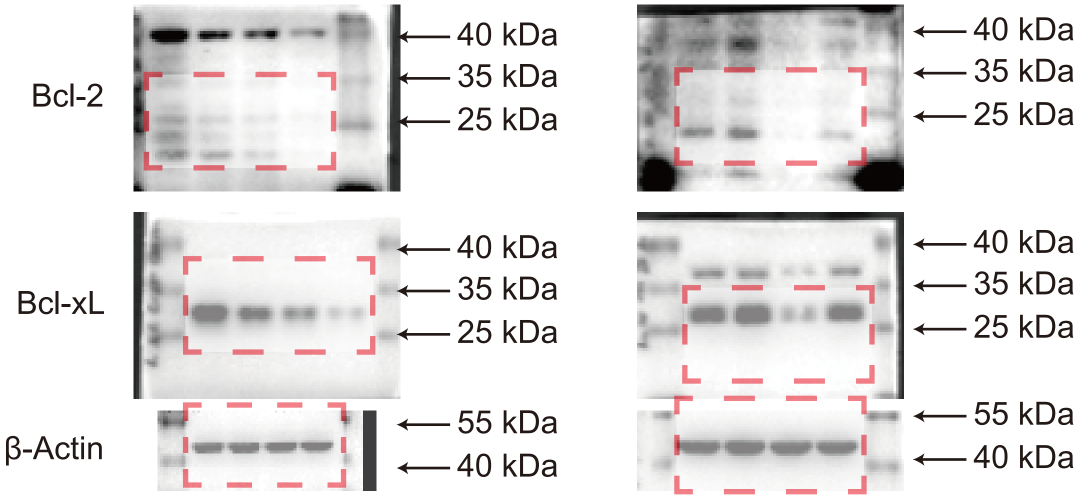

Figure 8B

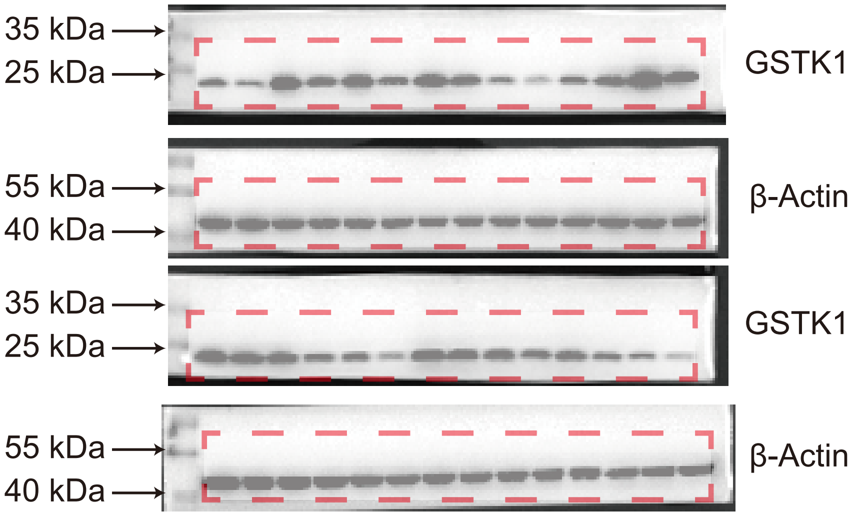

Figure 8E

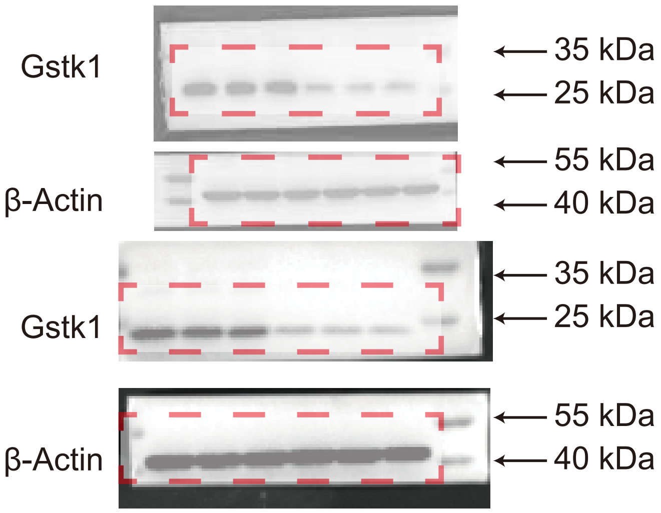

new Figure S1

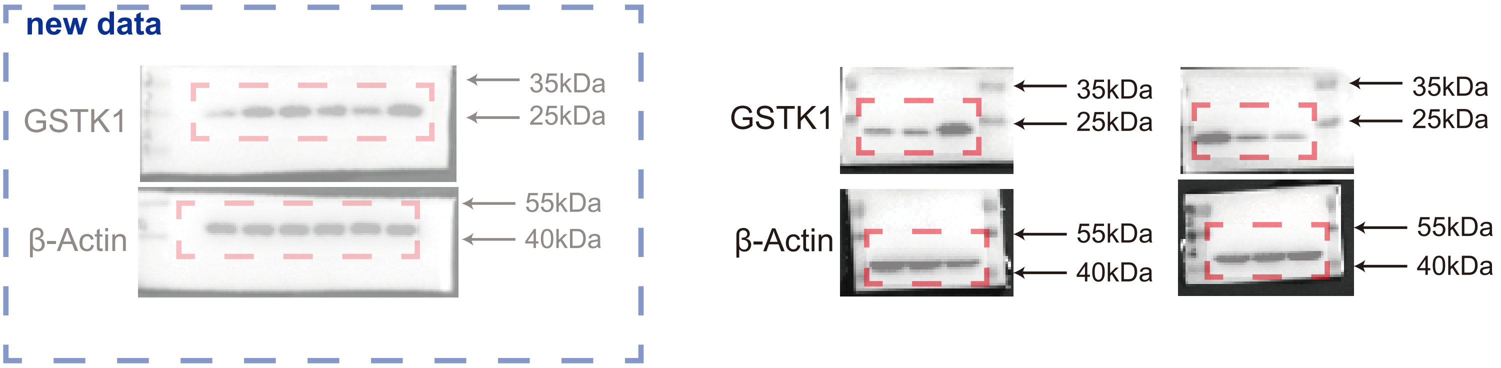

Figure S2

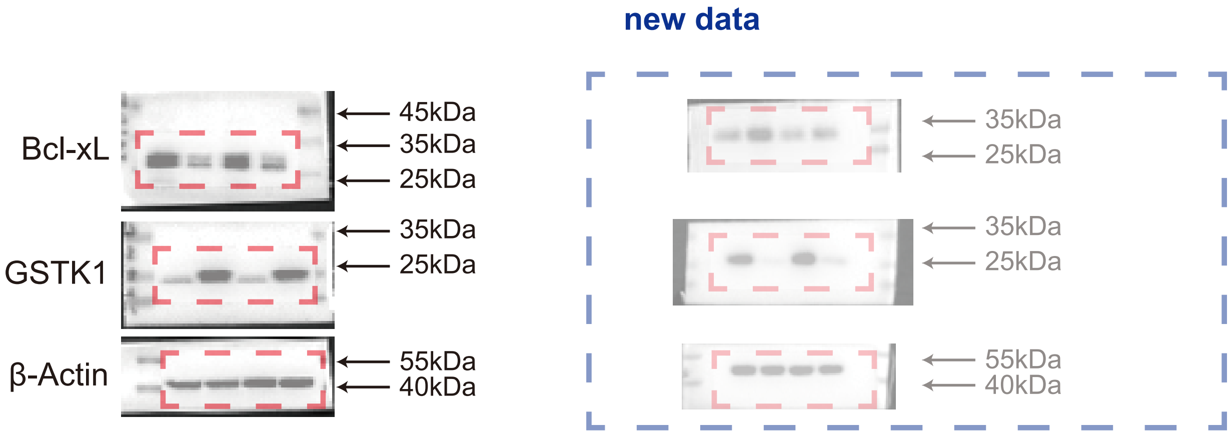

Figure S3H

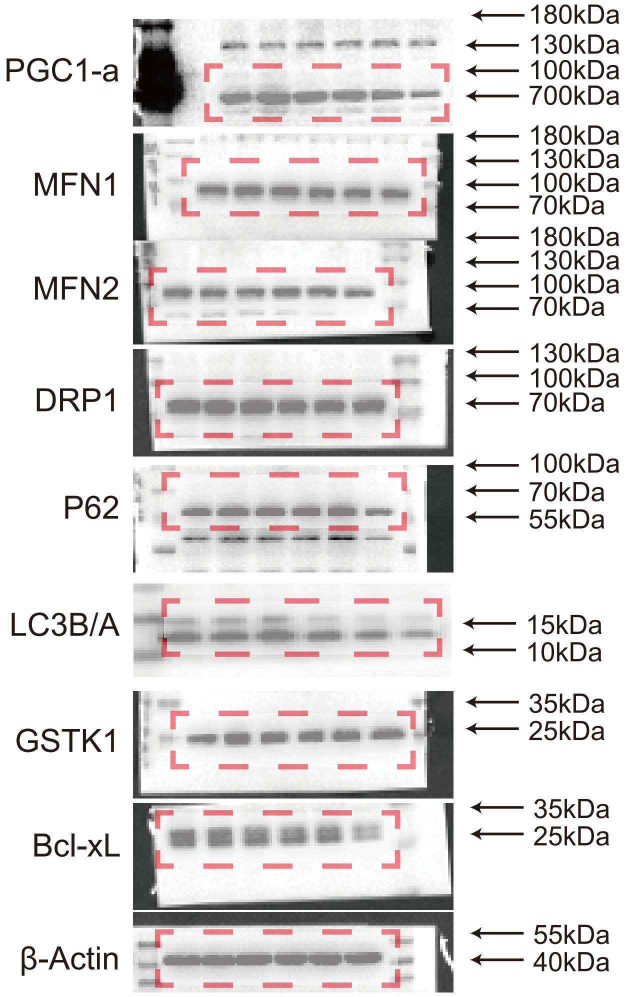

Figure S5E

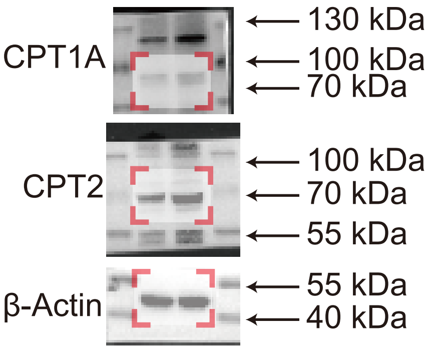

new data

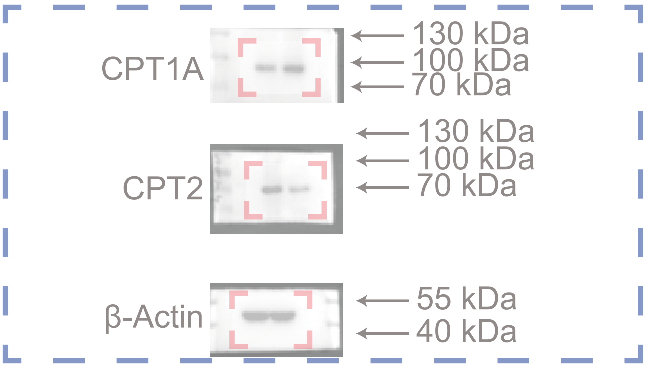

new data

Figure S6C&F

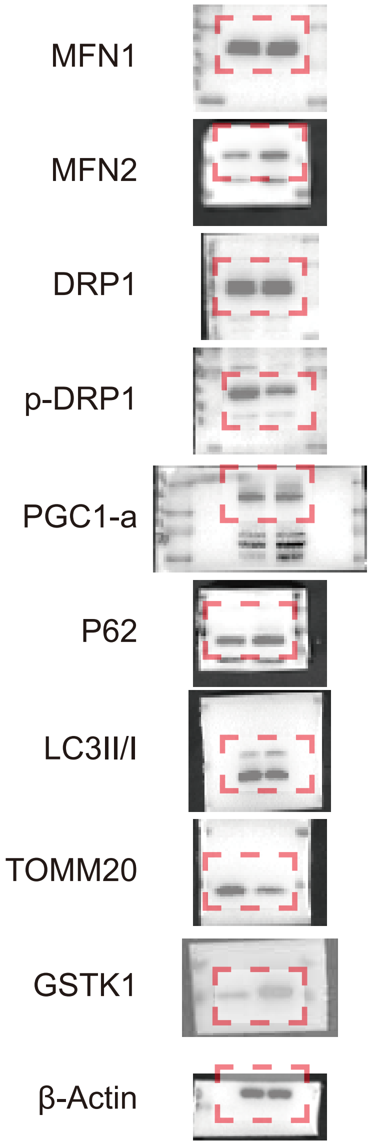

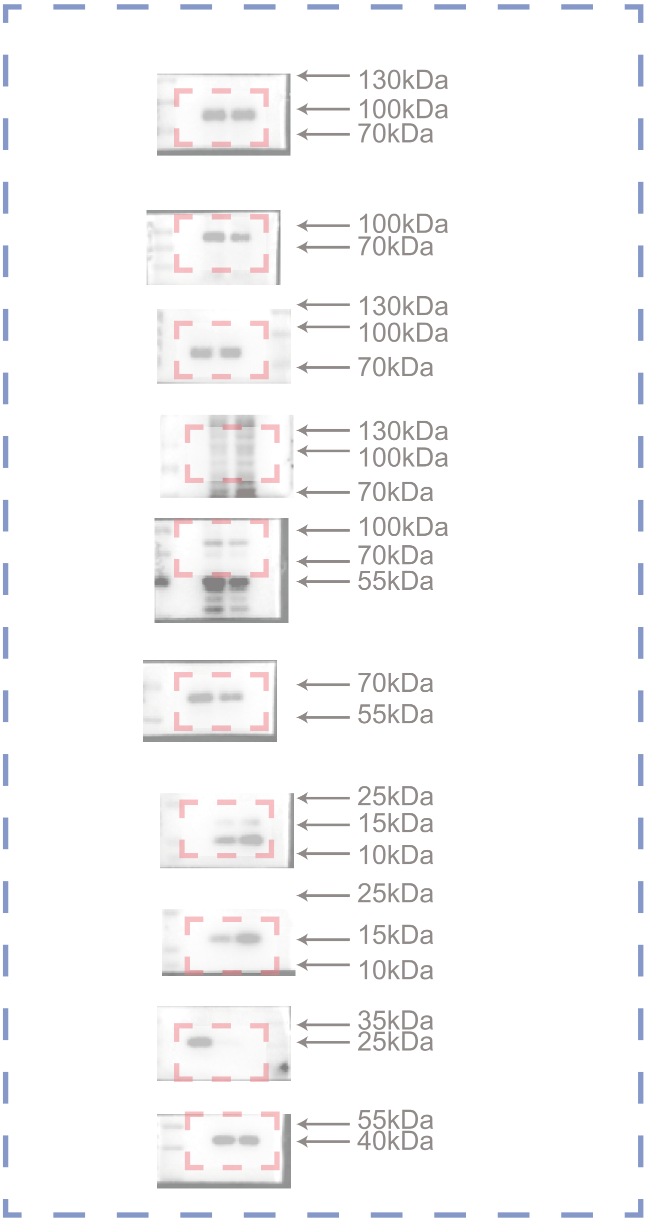

Figure S7A

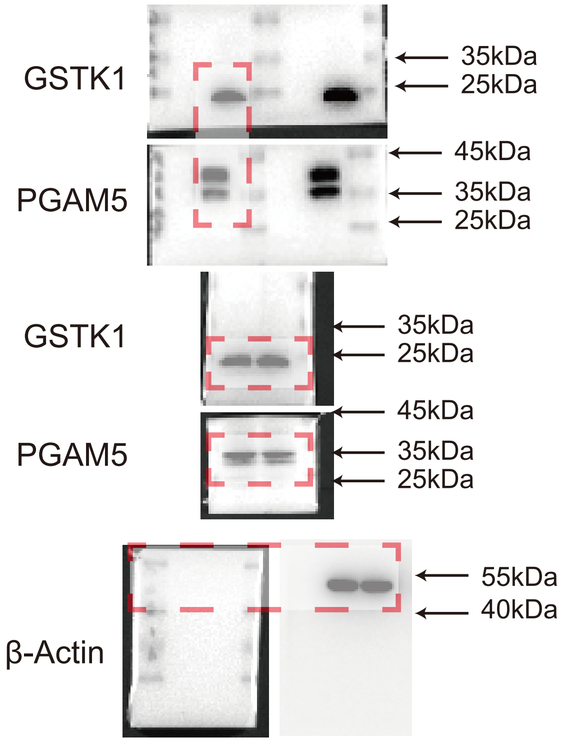

Figure S7B

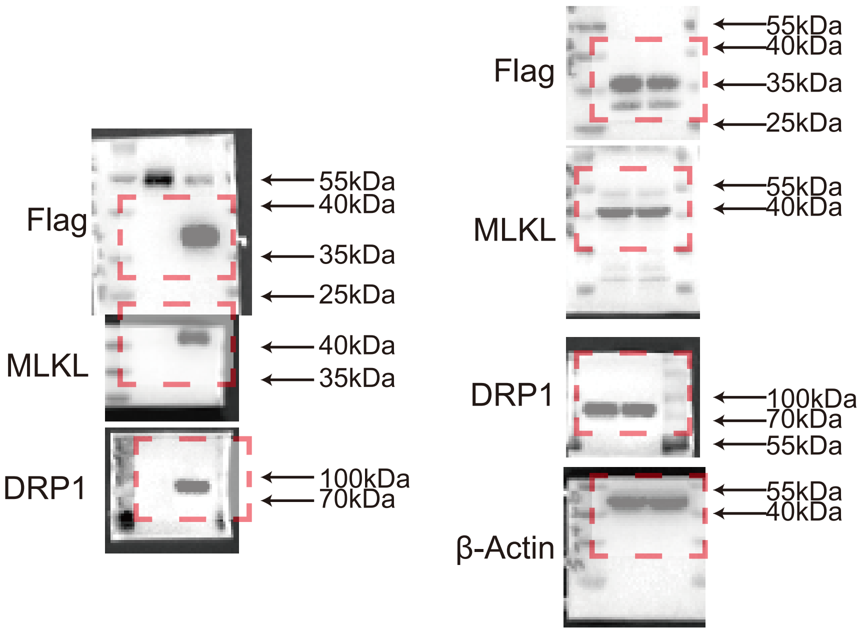

Figure S7E

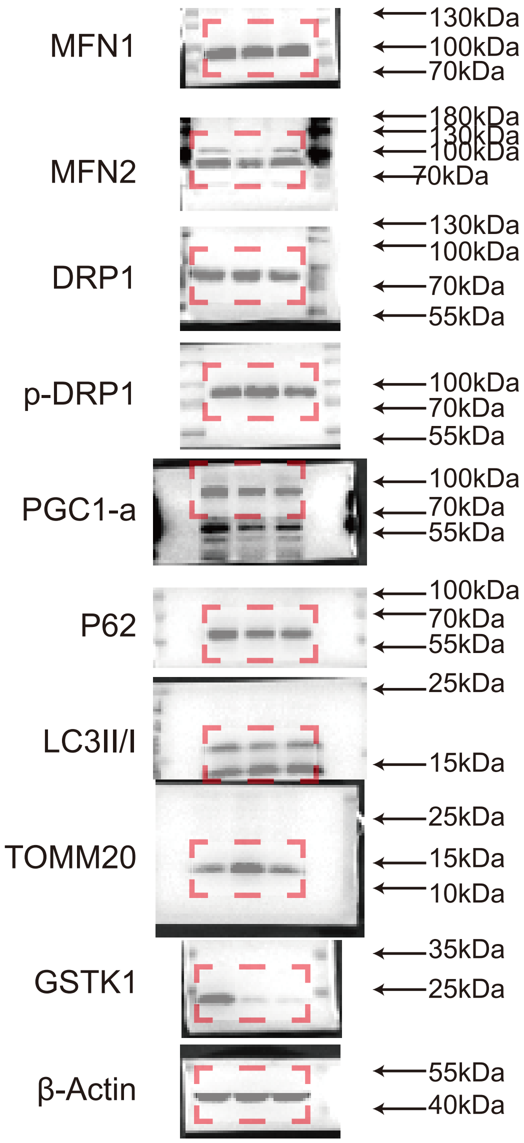

Figure S7G

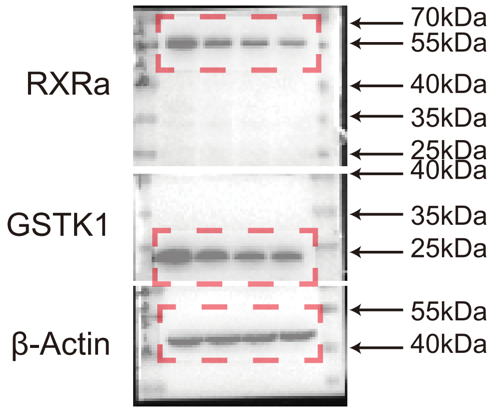

Supplement: Supplementary file 2 — Supplementary Material 2. [file 13046_2025_3580_MOESM2_ESM.pdf]
